# Socioeconomic characteristics of individuals who were and were not enrolled in two cardiovascular registry-based randomised controlled trials in Sweden: a descriptive cross-sectional study

**DOI:** 10.1136/bmjopen-2026-121876

**Published:** 2026-11-10

**Authors:** Anna Brigit Corinne Humphreys, Anita Berglund, Camila Olarte Parra, Javier Louro, Isabelle Munier, Bertil Lindahl, Stefan James, Ole Fröbert, Tomas Jernberg, Robin Hofmann, David Erlinge, Juho Härkönen, Anthony A. Matthews

**Affiliations:** 1 Unit of Epidemiology, Institute of Environmental Medicine, Karolinska Institute, Stockholm, Sweden; 2 Barcelona Institute for Global Health, Barcelona, Spain; 3 Department of Epidemiology and Evaluation, Hospital del Mar, Barcelona, Spain; 4 Department of Medical Sciences, Uppsala University, Uppsala, Uppsala County, Sweden; 5 Department of Cardiology, Faculty of Health, Örebro University Hospital, Örebro, Sweden; 6 Department of Clinical Medicine, Aarhus University Hospital, Aarhus, Denmark; 7 Department of Clinical Pharmacology, Aarhus University Hospital, Aarhus, Denmark; 8 Steno Diabetes Center Aarhus, Aarhus University Hospital, Aarhus, Denmark; 9 Department of Clinical Sciences, Danderyd University Hospital, Karolinska Institutet, Stockholm, Sweden; 10 Department of Clinical Science and Education, Division of Cardiology, Södersjukhuset, Karolinska Institutet, Stockholm, Sweden; 11 Department of Cardiology Clinical Sciences, Skåne University Hospital, Lund University, Lund, Sweden; 12 Department of Political and Social Sciences, European University Institute, Florence, Italy; 13 Department of Sociology, Stockholm University, Stockholm, Sweden

**Keywords:** Cardiac Epidemiology, Clinical Trial, EPIDEMIOLOGY, Health Equity

## Abstract

**Objectives:**

To describe socioeconomic differences between individuals who were and were not enrolled in two registry-nested trials, and to assess whether socioeconomic variables provide additional discriminatory value in predicting enrolment beyond routinely collected clinical data.

**Design:**

Descriptive cross-sectional study in Sweden using linked socioeconomic data.

**Setting:**

Two Swedish Web System for Enhancement and Development of Evidence-based Care in Heart Disease Evaluated According to Recommended Therapies (SWEDEHEART) registry-based cardiovascular randomised trials, Thrombus Aspiration in ST-Elevation myocardial infarction in Scandinavia (TASTE) (Swedish centres only) and Bivalirudin versus Heparin in ST-Segment and Non-ST-Segment Elevation Myocardial Infarction in Patients on Modern Antiplatelet Therapy (VALIDATE-SWEDEHEART).

**Participants:**

All individuals who were and were not enrolled in TASTE and VALIDATE-SWEDEHEART trials.

**Primary outcome measure:**

For both trials, baseline socioeconomic characteristics of individuals who were and were not enrolled were compared using standardised mean differences. Enrolment was then modelled using random forests based on (1) trial clinical variables, (2) socioeconomic variables and (3) both combined. The areas under the receiver operating characteristic curve (AUC) of the models were compared to assess the additional value of socioeconomic data in predicting enrolment.

**Results:**

In TASTE, 6841 of 11 436 potentially eligible individuals were enrolled, and in VALIDATE-SWEDEHEART, 5947 of 12 561 individuals were enrolled. Across both trials, enrolled individuals were more likely to be born in Sweden, be employed, be in a relationship, have a higher income and have a longer education duration than individuals who were not enrolled. The AUCs for enrolment models based solely on socioeconomic variables were similar to those using clinical variables. Adding socioeconomic variables to clinical models resulted in little or no improvement in AUC.

**Conclusions:**

Socioeconomic characteristics differed between individuals who were and were not enrolled in two registry-based randomised controlled trials. The limited improvement in the predictive performance after adding socioeconomic variables to clinical models likely reflects a strong correlation between socioeconomic status and clinical profile.

**Trial registration number:**

ClinicalTrials.gov identifiers: TASTE - NCT01093404, VALIDATE NCT02311231.

**STRENGTHS AND LIMITATIONS OF THIS STUDY**

- Strengths included the availability of detailed clinical and socioeconomic data on all individuals who were and were not enrolled in two trials.
- Many covariates could not be used in the clinical predictive models for enrolment due to substantial differences in the proportion of missing data between enrolled and non-enrolled individuals.
- These findings may not be representative of settings beyond Sweden or of clinical trials assessing interventions outside acute cardiac care.

## Introduction

 Individuals enrolled in cardiovascular randomised clinical trials often differ from the target population to which the results are intended to apply. A non-representative trial population may restrict the generalisability of trial results, while inequitable trial recruitment process may reduce engagement with, and trust in, the clinical trial process.^1 2^ Differences in demographic and clinical characteristics are well recognised: individuals who are enrolled in trials are often younger, more likely to be male, less ethnically diverse and have fewer comorbidities than those who are not enrolled,^3–7^ with these differences partly reflecting the restrictive eligibility criteria of many trials.^8–13^ Evidence on differences in socioeconomic characteristics—such as occupation, income or educational attainment—remains limited^14 15^; a gap compounded by the infrequent collection of these data in trials.^16^ Yet individuals with lower socioeconomic status often bear the greatest burden of ill health,^17–23^ and trial enrolment itself reflects a complex process shaped by multiple potential interrelated factors, many of which may be partly determined by socioeconomic circumstances.

Thrombus Aspiration in ST-Elevation myocardial infarction in Scandinavia (TASTE) and Bivalirudin versus Heparin in ST-Segment and Non-ST-Segment Elevation Myocardial Infarction in Patients on Modern Antiplatelet Therapy (VALIDATE-SWEDEHEART) are two cardiovascular randomised trials that were nested in the Swedish Web System for Enhancement and Development of Evidence-based Care in Heart Disease Evaluated According to Recommended Therapies (SWEDEHEART) registry.^24 25^ Of all potentially eligible individuals, 39% in TASTE and 52% in VALIDATE-SWEDEHEART were not enrolled. Results showed that individuals who were enrolled were younger on average, more likely to be male, and had a lower prevalence of comorbidities compared with those who were not enrolled.

There is, however, no empirical evidence comparing socioeconomic characteristics between those who were and were not enrolled in these trials. Such information would show the extent of socioeconomic disparities in trial enrolment, and could help inform public health interventions aimed at improving trial representativeness and ensuring equitable participation. Furthermore, evaluating whether socioeconomic factors add predictive value for enrolment over routinely collected clinical information can provide an additional layer of understanding of the complex processes that underlie trial enrolment.

Here, we first aim to describe differences in socioeconomic characteristics between individuals who were and were not enrolled in TASTE and VALIDATE-SWEDEHEART. Second, we aim to assess whether socioeconomic variables provide additional discriminatory value beyond routinely collected clinical data in predicting enrolment.

## Methods

Pseudonymised personal data were obtained from Swedish register holders after ethical approval and secrecy assessment. According to the General Data Protection Regulation and Swedish laws and regulations, sensitive personal data can only be made available for researchers who meet the legal requirements for access to such data. All individuals registered in SWEDEHEART are informed of their participation and have the right to opt-out, however, written informed consent is not needed. All analytical code is available at: https://github.com/Annahum/se.

### Patient and public involvement

Patients were not involved in the design, conduct or reporting of this research.

### Data sources

This descriptive cross-sectional study used SWEDEHEART, a nationwide healthcare quality registry that includes all individuals hospitalised in Sweden for acute coronary syndrome or undergoing coronary or valvular intervention.^26^ It is linked to national population registers, including the National Total Population Register, the National Patient Register, the National Prescribed Drug Register and the Swedish Longitudinal Integrated Database for Health Insurance and Labour Market Studies (LISA). LISA is a database composed of individuals who are registered in the Swedish Total Population Register on the 31 December each year.^27^ LISA collates information on a yearly basis from multiple sources, including the Education Register, the Register of Income and Taxation and the Swedish Social Security Agency. For this study, socioeconomic variables were obtained from LISA relating to migration, residential characteristics, family structure, education, occupation and income. Online supplemental table 1 contains the full list of included variables and their definitions. These variables were chosen a priori using a theory-informed approach to capture a comprehensive range of socioeconomic status dimensions relevant to the study aim. The last recorded values of the socioeconomic variables in the three years prior to the point of recruitment were used.

TASTE was a prospective multicentre open-label randomised trial that assessed the effect of percutaneous coronary intervention (PCI) with or without thrombus aspiration in individuals with ST-elevation myocardial infarction. Individuals were recruited between June 2010 and March 2013 across 29 centres in Sweden, one in Iceland and one in Denmark. Only individuals in Sweden are included in this study, of whom 11 436 were potentially eligible for enrolment and 6841 were enrolled (figure 1). It was not possible to identify and exclude the 15 individuals from the non-enrolled group who were erroneously initially enrolled despite not having ST-elevation myocardial infarction and who did not undergo PCI. VALIDATE-SWEDEHEART was also a prospective, open-label trial, with recruitment in Sweden alone. Individuals with myocardial infarction undergoing PCI and treated with a P_2_Y_12_ inhibitor between June 2014 and September 2016 were randomised to bivalirudin or heparin. Of the 12 561 potentially eligible individuals, 5947 were enrolled (figure 2). In this study, 59 individuals in the enrolled group were excluded for whom linked socioeconomic data were not available (including 49 who subsequently withdrew consent), leaving 5947 individuals for analysis.

**Figure 1:**
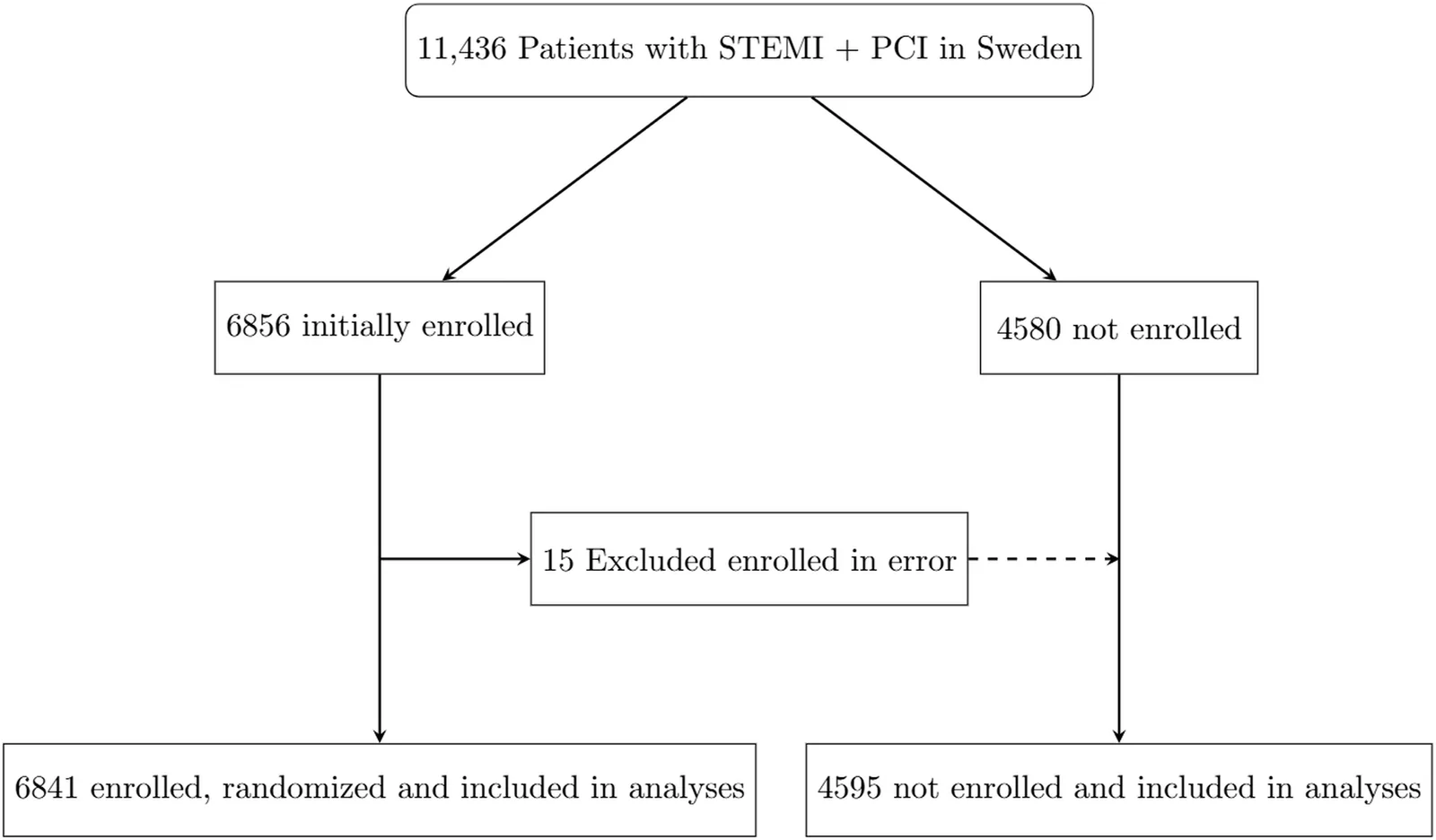
Flow chart showing individuals who were and were not enrolled in TASTE in Sweden. PCI, percutaneous coronary intervention; STEMI, ST-segment elevation myocardial infarction; TASTE, Thrombus Aspiration in ST-Elevation myocardial infarction in Scandinavia.

**Figure 2:**
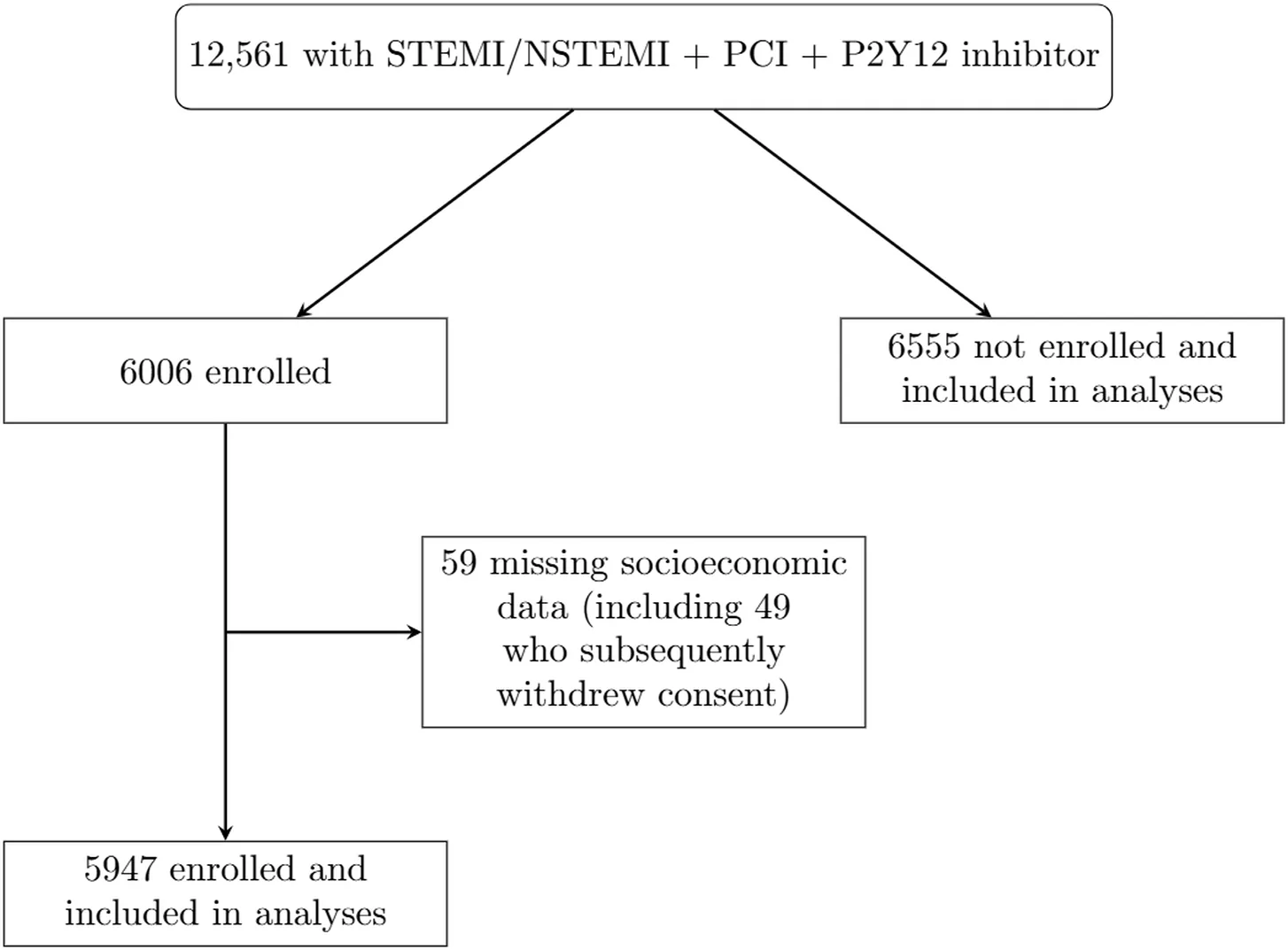
Flow chart showing individuals who were and were not enrolled in VALIDATE-SWEDEHEART. NSTEMI, non-ST elevantion myocardial infarction; PCI, percutaneous coronary intervention; STEMI, ST-segment elevation myocardial infarction; VALIDATE-SWEDEHEART, Bivalirudin versus Heparin in ST-Segment and Non-ST-Segment Elevation Myocardial Infarction in Patients on Modern Antiplatelet Therapy.

#### Analyses

All analyses described below were conducted in the TASTE and VALIDATE-SWEDEHEART data separately. All data contained both individuals who were and were not enrolled. Baseline socioeconomic characteristics of the enrolled and non-enrolled groups at the point of recruitment were compared using standardised mean differences (SMD). SMD quantify the magnitude of differences between groups. We considered SMD values exceeding 0.1 standard deviations to be indicative of meaningful differences. As older birth cohorts are known to have received lower levels of education, and to have a lower income and occupational status than younger birth cohorts, baseline characteristics were also stratified by age (<65 years and ≥65 years, to reflect the common age of retirement) and sex.^28 29^

A random forest was used to derive a predictive model for enrolment using the baseline clinical covariates available in the original trials.^24 25^ This non-parametric model was chosen as it can handle multidimensional data with multicollinearity or interactions between covariates and non-linear relationships.^30 31^ Due to the varying proportion of missing data between the enrolled and non-enrolled groups, only variables with less than 0.5% missing were included in the clinical model. Further details are provided in the Supplementary Appendix (including online supplemental tables 2-5). Clinical models included age, sex, previous PCI and previous coronary artery bypass grafting (TASTE only: warfarin use before PCI, heparin use before PCI and thrombolysis before PCI, and VALIDATE-SWEDEHEART only: myocardial infarction type). For the remaining missing data, a missing indicator was included for categorical variables. The only continuous variable included in the models, age, did not have any missing data. The random forest predictive model was validated through 10-fold cross-validation (full details in the online supplemental appendix).^32^ The overall ability of the random forest model to predict enrolment was evaluated using the area under the receiver operating characteristic curve (AUC).^33^

The same procedure was then used to derive a second random forest model for enrolment using only socioeconomic covariates (see online supplemental table 1 with individual disposable income included only in a categorical form). Socioeconomic covariate models additionally included age and sex. A third random forest model was derived using both socioeconomic and clinical covariates. The AUCs of the three models were compared with assess whether socioeconomic covariates provide additional predictive value beyond routinely available clinical covariates.

To understand the impact of missing data in the socioeconomic covariates, a sensitivity analysis was carried out using complete-case analysis.

## Results

### TASTE

The distribution of baseline characteristics between individuals who were and were not enrolled in TASTE can be seen in table 1, where those who were enrolled were more likely to be born in Sweden (86.2% vs 80.5%). There was marked variation in region of residence, with Stockholm and Linköping enrolling proportionally fewer than Uppsala, Lund-Malmö, Umeå and Gothenburg. Enrolled individuals were more likely to be married, cohabiting or in a partnership (58.3% vs 53.5%), have a longer education and were more likely to have studied a specialised subject. The median yearly disposable income of individuals who were enrolled was greater than those who were not (177 550 vs 1 51 030 Swedish krona (SEK), equating to about €16 509 vs €14 043 in March 2026) and individuals who were enrolled were less likely to be retired or unemployed (63.0% vs 71.7%).

**Table 1:** Baseline characteristics of individuals who were and were not enrolled in the TASTE trial

Variable	Enrolled	Not enrolled	Missing	SMD
N	6841	4595		
*Demographics and migration*				
Age (mean (SD))	66.5 (11.6)	68.8 (12.8)	0	0.193
Female	1746 (25.5)	1478 (32.2)	0	0.147
Country of birth			0	0.183
Sweden	5894 (86.2)	3697 (80.5)		
Other Nordic countries	348 (5.1)	243 (5.3)		
Europe excluding Nordics	365 (5.3)	383 (8.3)		
North and South America	38 (0.6)	22 (0.5)		
Other	196 (2.9)	250 (5.4)		
Immigration in the 3 years prior	30 (0.4)	47 (1.0)	0	0.069
Region of residence			12.5	0.276
Stockholm	769 (12.8)	707 (17.8)		
Uppsala	1677 (27.8)	938 (23.6)		
Linköping	600 (10.0)	650 (16.3)		
Lund-Malmö	1167 (19.4)	760 (19.1)		
Gothenburg	1299 (21.6)	620 (15.6)		
Umeå	515 (8.5)	307 (7.7)		
Degree of urbanisation			12.5	0.025
Most urbanised	1878 (31.2)	1280 (32.1)		
Mid urbanised	2650 (44.0)	1747 (43.9)		
Least urbanised	1499 (24.9)	955 (24.0)		
Moved municipality in last year	95 (1.6)	76 (1.9)	12.5	0.025
*Family structure*				
Family position			0.2	0.098
Single	2648 (38.8)	1988 (43.3)		
Single parent	195 (2.9)	145 (3.2)		
Married, cohabiting or in a partnership	3981 (58.3)	2453 (53.5)		
Divorce, separation or loss of partner in last 3 years	145 (2.1)	118 (2.6)	0.2	0.03
*Education*				
Education area			2.9	0.125
General education	2701 (40.3)	2023 (45.9)		
Social sciences	1111 (16.6)	676 (15.3)		
Science, technology, agriculture, health and social care	2485 (37.1)	1421 (32.2)		
Services	400 (6.0)	289 (6.6)		
Education length			1.6	0.107
Primary education ≤9 years	2512 (37.1)	1895 (42.3)		
Secondary education ≤3 years	3004 (44.4)	1810 (40.4)		
Tertiary education </>3 years	1255 (18.5)	772 (17.2)		
*Employment and income*				
Occupational status			0.4	0.191
Senior officials, managers and professionals	684 (10.0)	374 (8.2)		
Small business owners and farmers	363 (5.3)	191 (4.2)		
Supervisors, technicians and skilled manual workers	353 (5.2)	150 (3.3)		
Office employees and skilled workers in trade, services and care	553 (8.1)	272 (5.9)		
Other manual worker	566 (8.3)	310 (6.8)		
Retired or unemployed	4291 (63.0)	3279 (71.7)		
At least one day of unemployment last 3 years	568 (8.3)	335 (7.3)	0.2	0.038
Individual disposable income per hundred SEK	1775.5 (1304.3–2518.7)	1510.3 (1181.8–2274.0)	0.2	0.149
Individual disposable income per hundred SEK by quintile			0.2	0.266
1	1147 (16.8)	1135 (24.7)		
2	1252 (18.3)	1030 (22.5)		
3	1398 (20.5)	884 (19.3)		
4	1520 (22.3)	762 (16.6)		
5	1507 (22.1)	775 (16.9)		

SEK, Swedish krona; SMD, standardised mean differences; TASTE, Thrombus Aspiration in ST-Elevation myocardial infarction in Scandinavia.

### VALIDATE-SWEDEHEART

The distribution of baseline characteristics between individuals who were and were not enrolled in VALIDATE-SWEDEHEART can be seen in table 2. The results were similar to those of the TASTE trial, with individuals who were enrolled more likely to be born in Sweden (87.2% vs 76.7%), and Stockholm, Linköping and Umeå enrolling proportionally fewer than Uppsala, Lund-Malmö and Gothenburg. Similarly, individuals who were enrolled were more likely to be married, cohabiting or in a partnership (57.6% vs 53.7%), have a longer education and were more likely to have studied a specialised subject. The median yearly disposable income of individuals who were enrolled was greater than those who were not (199 930 vs 166 100 SEK, equating to about €18 590 vs €15 445), and individuals who were enrolled were less likely to be retired or unemployed (58.0% vs 71.8%).

**Table 2:** Baseline characteristics of individuals who were and were not enrolled in the VALIDATE-SWEDEHEART trial

Variable	Enrolled	Not enrolled	Missing	SMD
N	5947	6555		
*Demographics and migration*				
Age (mean (SD))	67.1 (11.0)	69.9 (12.1)	0	0.235
Female	1567 (26.3)	2000 (30.5)	0	0.092
Country of birth			0	0.298
Sweden	5186 (87.2)	5027 (76.7)		
Other Nordic countries	276 (4.6)	390 (5.9)		
Europe excluding Nordics	290 (4.9)	587 (9.0)		
North and South America	26 (0.4)	47 (0.7)		
Other	169 (2.8)	504 (7.7)		
Immigration in the 3 years prior	26 (0.4)	118 (1.8)	0	0.13
Region of residence			10.5	0.358
Stockholm	696 (13.2)	1371 (23.2)		
Uppsala	1487 (28.1)	1251 (21.2)		
Linköping	246 (4.6)	443 (7.5)		
Lund-Malmö	1150 (21.7)	1163 (19.7)		
Gothenburg	1213 (22.9)	939 (15.9)		
Umeå	499 (9.4)	737 (12.5)		
Degree of urbanisation			10.5	0.018
Most urbanised	1762 (33.3)	2017 (34.2)		
Mid urbanised	2296 (43.4)	2532 (42.9)		
Least urbanised	1233 (23.3)	1355 (23.0)		
Moved municipality in last year	107 (2.0)	106 (1.8)	10.5	0.017
*Family structure*				
Family position			0.3	0.078
Single	2344 (39.5)	2812 (43.1)		
Single parent	175 (2.9)	209 (3.2)		
Married, cohabiting or in a partnership	3419 (57.6)	3504 (53.7)		
Divorce, separation or loss of partner in last 3 years	122 (2.1)	188 (2.9)	0.3	0.053
*Education*				
Education area			3.1	0.136
General education	2041 (35.1)	2612 (41.5)		
Social sciences	1095 (18.8)	1081 (17.2)		
Science, technology, agriculture, health and social care	2314 (39.7)	2202 (35.0)		
Services	372 (6.4)	403 (6.4)		
Education length			1.5	0.139
Primary education ≤9 years	1836 (31.1)	2410 (37.6)		
Secondary education ≤3 years	2762 (46.8)	2746 (42.9)		
Tertiary education </>3 years	1310 (22.2)	1248 (19.5)		
*Employment and income*				
Occupational status			1.4	0.298
Senior officials, managers and professionals	741 (12.6)	507 (7.9)		
Small business owners and farmers	403 (6.8)	335 (5.2)		
Supervisors, technicians and skilled manual workers	300 (5.1)	197 (3.1)		
Office employees and skilled workers in trade, services and care	541 (9.2)	377 (5.9)		
Other manual worker	495 (8.4)	394 (6.1)		
Retired or unemployed	3421 (58.0)	4616 (71.8)		
At least 1 day of unemployment last 3 years	443 (7.5)	499 (7.6)	0.3	0.007
Individual disposable income per hundred SEK	1999.3 (1447.0–2917.7)	1661.0 (1316.7–2486.7)	0.3	0.068
Individual disposable income per hundred SEK by quintile			0.3	0.298
1	964 (16.2)	1529 (23.4)		
2	1038 (17.5)	1455 (22.3)		
3	1132 (19.1)	1361 (20.9)		
4	1398 (23.5)	1094 (16.8)		
5	1406 (23.7)	1086 (16.6)		

SEK, Swedish krona; SMD, standardised mean differences; VALIDATE-SWEDEHEART, Bivalirudin versus Heparin in ST-Segment and Non-ST-Segment Elevation Myocardial Infarction in Patients on Modern Antiplatelet Therapy.

### Subgroups

In both the TASTE and VALIDATE-SWEDEHEART trials, subgroup analyses of baseline characteristics by age and sex were broadly similar to the main analyses. However, differences in income and occupational status between the individuals who were and were not enrolled were in the same direction but larger in magnitude among younger individuals and men compared with the main analyses (online supplemental tables 6-13).

### Predictive model

For TASTE, the AUC was 0.57 (95% CI 0.56 to 0.59) for the predictive model for enrolment using only clinical covariates, 0.57 (95% CI 0.55 to 0.58) for the model using only socioeconomic characteristics and 0.59 (95% CI 0.57 to 0.60) for the model using both clinical and socioeconomic covariates (table 3). For VALIDATE-SWEDEHEART, the AUC was 0.62 (95% CI 0.61 to 0.63) using only clinical covariates, 0.60 (95% CI 0.58 to 0.61) using only socioeconomic covariates and 0.64 (95% CI 0.62 to 0.65) using both clinical and socioeconomic covariates. The sensitivity analyses were similar to the main results (online supplemental table 13).

**Table 3:** Random forest predictive model for enrolment

Covariates included	TASTE area under the ROC curve (95% CI)	VALIDATE-SWEDEHEART area under the ROC curve (95% CI)
Clinical	0.57 (0.56 to 0.59)	0.62 (0.61 to 0.63)
Socioeconomic	0.57 (0.55 to 0.58)	0.60 (0.58 to 0.61)
Clinical and socioeconomic	0.59 (0.57 to 0.60)	0.64 (0.62 to 0.65)

ROC, receiver operating characteristic; TASTE, Thrombus Aspiration in ST-Elevation myocardial infarction in Scandinavia ; VALIDATE-SWEDEHEART, Bivalirudin versus Heparin in ST-Segment and Non-ST-Segment Elevation Myocardial Infarction in Patients on Modern Antiplatelet Therapy.

## Discussion

Across both the TASTE and VALIDATE-SWEDEHEART trials, we show that individuals who were enrolled were more likely to be born in Sweden, be employed, have a higher disposable income, have completed a longer duration of education and be in a relationship compared with individuals who were not enrolled. Predictive models for enrolment based solely on socioeconomic variables performed similarly to models based solely on clinical variables, and combining clinical and socioeconomic variables yielded little or no improvement in performance.

In this study, both women and older individuals were less likely to be enrolled, which is consistent with previous evidence.^34–37^ This may reflect factors related to the trial itself (eg, restrictive eligibility criteria), individual-level factors (eg, engagement with and trust in the clinical trial process) and aspects of the broader healthcare environment (eg, which patients are approached for enrolment).^34–36^ It was therefore plausible that the socioeconomic differences seen between individuals who were and were not enrolled were secondary to age and sex. For example, the higher disposable income seen among individuals who were enrolled may reflect a smaller proportion of retired individuals in this group. However, in subgroup analyses of socioeconomic characteristics by age and sex, most differences remained of a similar magnitude to the main analyses, though some larger differences were observed in income and education predominantly for younger and male individuals. Together, the main results and subgroup analyses suggest that individuals who were enrolled are not representative of the target population with regards to socioeconomic characteristics.

The finding that socioeconomic variables did not substantially improve the predictive performance of clinical models should not be taken to imply that these factors lack importance. Rather, it likely reflects their shared underlying determinants, with health status often closely correlated with socioeconomic status.^18 19^ Socioeconomic variables may therefore act as proxies for broader differences in health literacy, healthcare-seeking behaviour or physician–patient interactions.

Comparing our results to other studies is challenging, as few randomised trials routinely record socioeconomic data or collect data on individuals who were not enrolled.^16 38^ One notable SWEDEHEART-based observational study aimed to assess the association of trial participation with socioeconomic status, clinical characteristics and outcomes.^14^ It included individuals who attended their 1-year rehabilitation visit after their index myocardial infarction, with participation in any trial ascertained at the 2-month and 1-year visits. Although a limited number of socioeconomic characteristics were available, univariate analysis showed that married individuals, individuals with a higher disposable income and those with a longer duration of education were more likely to have participated in a trial, which is in line with the findings of this study. Here, we have focused on cardiovascular trials, acute settings and linked population registries. Outside these settings, studies with limited sample sizes which are often integrated within existing cohorts have found broadly similar results.^39–41^

When data on non-enrolled individuals are available, a challenge arises due to the varying reasons for non-enrolment. In TASTE and VALIDATE-SWEDEHEART, many individuals in the non-enrolled group were ineligible due to not fulfilling a clinical criterion or lack of consent while some were not asked to enrol or were excluded for reasons not listed in the trial protocols. Non-enrolment may therefore result from an individual’s decision to decline participation, which may reflect broader levels of trust in and engagement with the healthcare system. It may also arise from an inability to provide informed consent, for example, because of clinical condition or language barriers. Finally, non-enrolment may stem from differences in recruitment practices and trial engagement at the clinician, hospital and regional levels. This may in part explain the marked variation in enrolment observed across regions of residence in this study. As levels of socioeconomic deprivation vary both between and within regions in Sweden,^42^ socioeconomic status alone is unlikely to explain this finding. It was not possible to identify otherwise clinically eligible individuals who were not asked to participate.

The varied reasons for non-enrolment raise the question of whether these individuals would qualify for the target population, where consent would not be an inclusion criterion. Here, given the reasons for exclusion, it is likely that a substantial portion of the non-enrolled group would be in the target population.

A key strength of this study was the availability of data on all individuals who were and were not enrolled in two large trials, facilitated by the integration of the trials within a registry. A wide breadth of individual-level socioeconomic variables was available through linkage to population registers, providing comprehensive information on multiple dimensions of socioeconomic status. Many papers assessing socioeconomic status in settings largely outside of trials rely on aggregate, often geographically determined, deprivation scores^43 44^ or have limited socioeconomic variables such as income, education and relationship status.^14 20 22 23^

A key limitation was the inability to include many covariates—some prognostic—in the clinical predictive models for enrolment due to substantial differences in the proportion of missing data between enrolled and non-enrolled groups. The predictive performance of the clinical model may therefore be lower than expected, given the absence of potentially important clinical covariates. Both TASTE and VALIDATE investigated point interventions delivered in acute cardiac settings. Additionally, healthcare in Sweden is predominantly tax-funded and freely available.^45^ Therefore, both the proportion of individuals who were enrolled^38 46–48^ and the observed socioeconomic differences may not be representative of other countries or clinical contexts outside of acute cardiac interventions.

In summary, we found differences in multiple socioeconomic domains between individuals who were and were not enrolled in two cardiovascular registry-based randomised trials. The limited improvement in predictive performance for enrolment from the addition of socioeconomic variables is likely a result of close correlation between socioeconomic and health status. Recognising differences is an important step in order to improve the representativeness of trials. Routine documentation of socioeconomic characteristics in trials will enable the equity in recruitment to trials to be monitored and help to ensure all individuals have the opportunity to participate in clinical research.

## Supplementary material

10.1136/bmjopen-2026-121876 online supplemental file 1

## Data Availability

Data may be obtained from a third party and are not publicly available.
