# Supplementary material for: Socioeconomic characteristics of individuals who were and were not enrolled in two cardiovascular registry-based randomised controlled trials in Sweden: a descriptive cross-sectional study
Source: BMJ Open. 2026 Nov 10;16(11):e121876. doi: 10.1136/bmjopen-2026-121876 (PMC13756278; doi:10.1136/bmjopen-2026-121876)
Supplement: Supplementary file 1 [file bmjopen-2026-121876.reviewer_comments.pdf]

PEER REVIEW HISTORY

BMJ Open publishes all reviews undertaken for accepted manuscripts. Reviewers are asked to complete a checklist review form and are provided with free text boxes to elaborate on their assessment. These free text comments are reproduced below.

ARTICLE DETAILS

Title (Provisional)

Socioeconomic characteristics of individuals who were and were not enrolled in two cardiovascular registry-based randomised controlled trials in Sweden: a descriptive cross-sectional study

Authors

Humphreys, Anna Brigit Corinne; Berglund, Anita; Olarte Parra, Camila; Louro, Javier; Munier, Isabelle; Lindahl, Bertil; James, Stefan K; Frobert, Ole; Jernberg, Tomas; Hofmann, Robin; Erlinge, David; Härkönen, Juho; Matthews, Anthony

---

VERSION 1 - REVIEW

**Reviewer**	**1**
**Name**	**Dewi, Ayu Putu Madri**
**Affiliation**	**Amsterdam UMC Location AMC, Epidemiology and Data Science**
**Date**	**28-May-2026**
**COI**	**NA**

Dear authors,

To improve the quality of your manuscript, kindly consider the following comments:

- Please provide the study objective and design in the abstract.

- Please clarify in the Background why representativeness or equity of socioeconomic characteristics matters in relation to enrolment in clinical trials.

- In the Introduction section, I find that the study aim is not clearly stated, I presume the whole aim is to assess whether certain groups systematically underrepresented.

- The first aim is unclear. It mentions assessing socioeconomic characteristics, but it is not specified how these characteristics will be assessed. I presume the intended first aim is to assess the differences in socioeconomic characteristics between individuals who enrolled in, and those who were unwilling to enrol in, two clinical trials in Sweden.

- For the second aim, you mention assessing the additional collective value of socioeconomic variables; what do you mean? I presume, the intended second aim seems to assess whether socioeconomic characteristics and clinical characteristics predict participation in the two clinical trials in Sweden.

- Please clarify in the method section, what is the study design?

- Please clarify the rationale for using predictive modelling in this study. Since participation in RCTs should be voluntary, you wouldn't predict or expect for individual with certain characteristics to participate as they're voluntary. Hence, I find it is unclear whether the purpose of the analysis is to explain differences in representativeness between enrolled and non-enrolled individuals in RCTs or to develop a practically useful prediction model.

- Please explain which variables were included in the analysis. Clinical characteristics variable were not clearly described. I presume these included BMI, smoking status, diabetes, previous stroke, etc., based on the code available on GitHub, but these should also be stated in the manuscript.

- Table 1 (TASTE trial): overall, most SMD values are low, with only a few moderate imbalances (age, country of birth, employment and income, and individual disposable income per hundred SEK by quintile). Why are these interpreted as 'clear socioeconomic differences'? Also, since the trial only included individuals from Sweden, imbalance in "country" is not surprising. Similar comments apply to Table 2.

- Tables 1 and 2 report age, is this all age? Because the Analyses section mentioned baseline characteristics stratified by age.

- Lines 14–17, page 8 of 81: exclusion of variables with missing data >0.5% is confusing. Differential missingness between groups and the overall amount of missing data are not the same and require further clarification. I presume, you excluded variables with substantial differential missingness (>0.5%) between enrolled and non-enrolled participants to minimise selection bias and instability in the prediction model.

- Line 47, page 17 of 81: the statement regarding "clear socioeconomic differences" appears overstated. Most SMD values indicate only low to moderate imbalance and should be interpreted cautiously.

- Supplementary methods - model development and validation: the validation strategy is difficult to follow. Please clarify the rationale for combining bootstrap resampling with 10-fold cross-validation.

- It is unclear at which stage preprocessing and missing value handling were performed relative to fold partitioning. If preprocessing occurred before splitting, of course data leakage may have occurred.

- The statement "the data was formatted to address missing values" is too vague for reproducibility. Please specify the preprocessing or imputation methods used.

- Prediction performance assessment appears limited to AUC only. Ideally, calibration assessment should also be included. I find this assessment is limited.

- Limitations section (lines 112–122, page 3 of 81): I do not see analyses assessing the relationship between socioeconomic and clinical characteristics. I think an important limitation is that this study may not be generalisable, as it only involved two trials in Sweden.

Reproducibility

- Analytical code is available via GitHub, but no data are available, so I cannot assess the reproducibility of the findings.

- Since the data were already pseudonymised, data sharing may still be feasible.

- The code appears partially reproducible. Bootstrap and seeds are provided, which is ok, but there are inconsistencies such as handling of lopnr (removed and later reused). Please double check whether the workflow is fully reproducible. Alternatively please get an independent CODECHEK.

Transparency

- Please provide links to study registration and/or study protocol, if available.

- Please provide a completed reporting checklist such as STROBE or TRIPOD.

- The coding workflow is scripted and annotated, which is appreciated.

**Reviewer**	**2**
**Name**	**Birhanu, Mulugeta**
**Affiliation**	**Monash Health, Medicine**
**Date**	**11-Aug-2026**
**COI**	**NA**

Reviewer comments

The paper entitled “Differences in socioeconomic characteristics of individuals who are and are not enrolled in cardiovascular registry based randomised controlled trials” has the potential to contribute to the existing body of literature. This is a well written manuscript. However, substantial revision is required regarding the methods, interpretation of the findings, and discussion of the potential implications and limitations.

Major comments:

The authors should provide further clarification and discuss regarding the reasons why eligible individuals did not participate in the TASTE and VALIDATE-SWEDEHEART trials. Is that because of restrictive eligibility criteria, reduce engagement, language barrier, concern or

lack of trust in clinical trial, consent, or other reason? This information would help readers to better understand the reasons for the differences.

Even though the authors preferred to analyse the Sweden data, there is still a variation in region of residence. The authors should provide further discussion of the potential reasons for these regional differences. For example, could these differences be explained by variation in the sociodemographic characteristics of the regions, differences in access to participating trial centres, or other contextual factors.

The author provided the SMD rather than P-values for comparison between groups. For the readers, providing clear explanation on the interpretation of the SMDs would essential.

In the summary, the authors mentioned that as they found clear socioeconomic differences between individuals who were and were not enrolled in the trials but the definition for socioeconomic is broad and should be interpreted with caution.

The authors also need to discuss the potential reasons of why women are less likely to be involved in trials than men? Is that because of eligibility, comorbidity, socioeconomic circumstances, caregiving responsibilities, health seeking behaviour, lack of transport, willingness, structural and social factors, and others? And the result also did not include information regarding the discrepancies between men and women.

Statistical analysis: The variables included in the clinical covariates, socioeconomic characteristics, and both clinical and socioeconomic covariate models are not clearly specified in the manuscript. The authors should provide the detailed list of variables included in each model and explain how these variables were selected. In particular, it would be helpful to clarify whether the variables were selected a priori based on clinical or theoretical considerations, based on previous literature, or using a data-driven approach.

I suggest the to provide the predictive model performance results in Table in the main manuscript as they did for sensitivity analysis in the online supplement.

The generalisability of the finding to other trial is quite limited and should be discussed more explicitly.

Minor comments

There are a relatively large number of Tables in the manuscript. I suggest consolidating tables where possible to improve readability and reduce redundancy. For example, Supplementary Tables 4 and 5 could be combined into a single Table.

Minor comments

Result page 9 of 81 (line 16): I suggest removing the phrase “have no occupational status or” as it is confusing to readers.

General comments:

The authors provided an interesting and important area of research but the methods, result and discussion require major revision. The implication or the interpretation of the findings needs some caution.

VERSION 1 - AUTHOR RESPONSE

Reviewer: 1
Ms. Ayu Putu Madri Dewi, Amsterdam UMC Location AMC
Comments to the Author:
Dear authors,

To improve the quality of your manuscript, kindly consider the following comments:

- Please provide the study objective and design in the abstract.

  Response - We have amended the abstract as follows (page 2):

  "Background: Individuals enrolled in cardiovascular randomised controlled trials are often not representative of the target population with respect to age, sex, ethnicity, and comorbidity profile. However, the extent of these differences for socioeconomic characteristics remains largely unquantified. The aim of this study was first to describe socioeconomic differences between individuals who were and were not enrolled in two registry-nested trials, and second, to assess the additional collective value of socioeconomic variables in predicting enrolment beyond routinely collected clinical data.

  Methods: In this descriptive cross-sectional study, ~~the~~ baseline socioeconomic characteristics of individuals who were and were not enrolled in two SWEDEHEART registry-based cardiovascular randomised trials, TASTE (Swedish centres only) and VALIDATE-SWEDEHEART, were compared using standardised mean differences."

- Please clarify in the Background why representativeness or equity of socioeconomic characteristics matters in relation to enrolment in clinical trials.

  Response - We have amended the text as follows (page 4):

  "A non-representative trial population may ~~both~~ restrict the generalisability of trial results, ~~and~~ whilst inequitable trial recruitment may reduce engagement with, and trust in, the clinical trial process (1,2)."

- In the Introduction section, I find that the study aim is not clearly stated, I presume the whole aim is to assess whether certain groups systematically underrepresented.

  Response - This is a descriptive paper in which we aimed to describe how socioeconomic characteristics such as income or education differed between individuals who were and were not enrolled in two registry-nested trials. We have amended the text as follows (page 6):

"Here, we first aim to describe ~~the~~ differences in socioeconomic characteristics between ~~of~~ individuals who were and were not enrolled in TASTE and VALIDATE-SWEDEHEART."

- The first aim is unclear. It mentions assessing socioeconomic characteristics, but it is not specified how these characteristics will be assessed. I presume the intended first aim is to assess the differences in socioeconomic characteristics between individuals who enrolled in, and those who were unwilling to enrol in, two clinical trials in Sweden.

Response - Our aim was to assess how socioeconomic characteristics differed between individuals who were and were not enrolled in two trials. In the methods, we describe that we use standardised mean differences to compare the socioeconomic characteristics.

However, the non-enrolled group is not just composed of individuals who decline to participate. In the discussion, we explore the composition of the non-enrolled group as a limitation to this paper. The non-enrolled group includes individuals who declined to consent, but also those who could not consent, were not asked or did not fulfil eligibility criteria.

- For the second aim, you mention assessing the additional collective value of socioeconomic variables; what do you mean? I presume, the intended second aim seems to assess whether socioeconomic characteristics and clinical characteristics predict participation in the two clinical trials in Sweden.

Response - Our aim was not to determine whether clinical or socioeconomic characteristics predict enrolment per se, but rather to assess the additional predictive value of socioeconomic characteristics beyond routinely available clinical covariates. We did this to provide an additional layer of understanding to the complex processes that underlie enrolment.

- Please clarify in the method section, what is the study design?

Response - We have clarified as follows (page 5):

"This descriptive cross-sectional study used SWEDEHEART, ~~is~~ a nationwide healthcare quality registry that includes all individuals hospitalised in Sweden for acute coronary syndrome or undergoing coronary or valvular intervention (26)."

- Please clarify the rationale for using predictive modelling in this study. Since participation in RCTs should be voluntary, you wouldn't predict or expect for individual with certain characteristics to participate as they're voluntary. Hence, I find it is unclear whether the purpose of the analysis is to explain differences in representativeness between enrolled and non-enrolled individuals in RCTs or to develop a practically useful prediction model.

Response - As you describe, participation in a trial is voluntary. However, there are many underlying reasons why an individual may not enrol such as the clinician not approaching the patient, language barriers making the consent process challenging,

or the patient being too ill to consent. Many of these reasons likely relate to socioeconomic status. Our goal was not to build a prediction model that can be used in other settings. Rather, we used a prediction model for descriptive purposes, and to give us the opportunity to assess whether socioeconomic characteristics provide any additional value beyond clinical covariates in understanding who will enrol. We have amended the discussion as follows (paged 16):

"When data on non-enrolled individuals are available, a challenge arises due to the varying reasons for non-enrolment. In TASTE and VALIDATE-SWEDEHEART, many individuals in the non-enrolled group were ineligible due to not fulfilling a clinical criterion or lack of consent ~~(either due to declining or being unable to provide it),~~ while some were not asked to enrol or were excluded for reasons not listed in the trial protocols. Non-enrolment may therefore result from an individual's decision to decline participation, which may reflect broader levels of trust in and engagement with the healthcare system. It may also arise from an inability to provide informed consent, for example because of clinical condition or language barriers. Finally, non-enrolment may stem from differences in recruitment practices and trial engagement at the clinician, hospital, and regional levels. This may in part explain the marked variation in enrolment observed across regions of residence in this study. As levels of socioeconomic deprivation vary both between and within regions in Sweden (42), socioeconomic status alone is unlikely to explain this finding. It was not possible to identify otherwise clinically eligible individuals who were not asked to participate.

The varied reasons for non-enrolment raise~~s~~ the question of whether these individuals would qualify for the target population, where consent would not be an inclusion criterion. Here, given the reasons for exclusion, it's likely that a substantial portion of the non-enrolled group would be in the target population."

- Please explain which variables were included in the analysis. Clinical characteristics variable were not clearly described. I presume these included BMI, smoking status, diabetes, previous stroke, etc., based on the code available on GitHub, but these should also be stated in the manuscript.

Response - All socioeconomic covariates used (including their definition, form and source) can be found in Supplementary Table One. Clinical covariates are listed in the main text. We have amended the text as follows for clarity (page 6):

"For this study, ~~additional~~ socioeconomic variables were obtained from LISA relating to migration, residential characteristics, family structure, education, occupation and income. ~~(see~~ Supplementary Table 1 ~~for~~ contains the full list of included variables and their definitions ~~list)~~. These variables were chosen a priori using a theory-informed approach to capture a comprehensive range of socioeconomic status dimensions relevant to the study aim. The last recorded values of the socioeconomic variables in the three years prior to the point of recruitment were used".

Page 7:

"Due to the varying proportion of missing data between the enrolled and non-enrolled groups, only variables with less than 0.5% missing were included in the clinical model. Further ~~full~~ details are provided in the Supplementary Appendix (including Supplementary Tables 2-5). Clinical models included age, sex, previous PCI and previous coronary artery bypass grafting (TASTE only: warfarin use before PCI, heparin use before PCI, and thrombolysis before PCI, and VALIDATE-SWEDEHEART only: myocardial infarction type)."

Page 7:

"The same procedure was then used to derive a second random forest model for enrolment using only socioeconomic covariates (see Supplementary Table 1 with individual disposable income included only in a categorical form). Socioeconomic covariate models additionally included ~~with~~ age and sex. ~~and finally~~ A third random forest model was derived using both socioeconomic and clinical covariates."

- Table 1 (TASTE trial): overall, most SMD values are low, with only a few moderate imbalances (age, country of birth, employment and income, and individual disposable income per hundred SEK by quintile). Why are these interpreted as 'clear socioeconomic differences'? Also, since the trial only included individuals from Sweden, imbalance in "country" is not surprising. Similar comments apply to Table 2.

Response - We considered standardised mean differences greater than 0.1 to be indicative of a clinically meaningful difference.

You're right in that we restricted the studies to individuals recruited in Sweden (VALIDATE-SWEDEHEART was only conducted in Sweden). However, for this study we present the country of birth, which we believe is an important characteristic.

We have amended the text as follows (page 6):

"Standardised mean differences (SMD) quantify the magnitude of differences between groups. We considered SMD values exceeding 0.1 standard deviations to be indicative of meaningful differences."

- Tables 1 and 2 report age, is this all age? Because the Analyses section mentioned baseline characteristics stratified by age.

Response - Tables 1 and 2 show the socioeconomic characteristics (in addition to age and sex) for individuals who were and were not enrolled in TASTE and VALIDATE-SWEDEHEART. In the results section, we have not described the differences in age and sex as these are described in the introduction.

We additionally conducted subgroup analyses where we stratified by age and sex as older birth cohorts are known to have received less education, lower income and a lower occupational status than younger birth cohorts (Supplementary Tables 5-12).

- Lines 14–17, page 8 of 81: exclusion of variables with missing data >0.5% is confusing. Differential missingness between groups and the overall amount of missing data are not the same and require further clarification. I presume, you excluded variables with substantial differential missingness (>0.5%) between enrolled and non-enrolled participants to minimise selection bias and instability in the prediction model.

  Response - We excluded clinical covariates which were missing more than 0.5%. In the Supplementary Appendix, the distribution of missingness between enrolled and non-enrolled individuals for both clinical and socioeconomic variables can be seen. We found that clinical variables were much more likely to be missing for non-enrolled than enrolled individuals, but this did not happen for socioeconomic covariates. In the Supplementary Appendix we further justify our reasoning for including clinical covariates with less than 0.5% missing. For example, we could not be sure of the temporal order of the clinical covariates being recorded – it is possible that some may have been recorded after enrolment, and thus directly influenced by the decision to enrol. We therefore only included variables that had a low proportion of missing data. In the limitations section of the discussion, we discuss how this decision may have resulted in an imperfect clinical model.

- Line 47, page 17 of 81: the statement regarding “clear socioeconomic differences” appears overstated. Most SMD values indicate only low to moderate imbalance and should be interpreted cautiously.

  Response - We considered standardised mean difference values of >0.1 to be meaningfully different. We have amended the text to clarify this point (page 6):

  “Baseline socioeconomic characteristics of the enrolled and non-enrolled groups at the point of recruitment were compared using standardised mean differences. Standardised mean differences quantify the magnitude of differences between groups. We considered SMD values exceeding 0.1 standard deviations to be indicative of meaningful differences.”

- Supplementary methods - model development and validation: the validation strategy is difficult to follow. Please clarify the rationale for combining bootstrap resampling with 10-fold cross-validation.

  Response - Bootstrapping was used to estimate point estimates and corresponding confidence intervals, whilst ten-fold cross validation was used to test and validate the random forest models. We have amended the supplementary appendix as follows:

  “To estimate the area under the receiver operating characteristic curve (AUC) for the random forest model predicting enrolment, we used the *ranger* package in R with parallel processing across multiple central processing units

to reduce computation time. Bootstrapping was used to estimate the point estimate and derive corresponding 95% confidence intervals."

- It is unclear at which stage preprocessing and missing value handling were performed relative to fold partitioning. If preprocessing occurred before splitting, of course data leakage may have occurred.

  Response - Thank you for identifying this issue. We bootstrapped the data by resampling with replacement and applied the missing-data handling procedures before the ten-fold cross-validation. While preprocessing prior to fold allocation could potentially introduce information leakage when continuous variables are categorised using data-derived cut-points, this is not relevant to our analysis because all model covariates were categorical except age, which had no missing data and was therefore not categorised. Consequently, we do not believe that meaningful data leakage occurred as a result of the preprocessing steps.

  We have clarified in the manuscript (page 7):

  "For the remaining missing data, a missing indicator was included for categorical variables. The only continuous variable included in the models, age, did not have any missing data." ~~,and continuous variables were categorised to also include a missing indicator.~~

  This has also been clarified in the R script (func_randomforest_cv_10fold_hyper.R, line 78).

- The statement "the data was formatted to address missing values" is too vague for reproducibility. Please specify the preprocessing or imputation methods used.

  Response - The methods section in the manuscript describes both how missing data was dealt with in the main analysis and in the sensitivity analysis.

  We have added further detail to the Supplementary Appendix for clarity "Within each bootstrap, the data ~~was~~ were formatted to address missing values (either to include a missing indicator, or for the sensitivity analysis, a complete case analysis) and then 10-fold cross-validation was applied."

- Prediction performance assessment appears limited to AUC only. Ideally, calibration assessment should also be included. I find this assessment is limited.

  Response - We thank the reviewer for this important point regarding model performance. The primary objective of our predictive model was to assess hypothesis-driven relative model improvement, specifically evaluating whether socioeconomic covariates provide incremental discriminatory power beyond clinical factors. To address this specific aim, we focused our evaluation on measures of discrimination (AUC). Because our goal was to quantify the relative contribution of these covariates rather than to report absolute predicted probabilities for individual

risk stratification or external decision support tools, we did not include formal calibration assessments.

- Limitations section (lines 112–122, page 3 of 81): I do not see analyses assessing the relationship between socioeconomic and clinical characteristics. I think an important limitation is that this study may not be generalisable, as it only involved two trials in Sweden.

  Response - The relationship between clinical characteristics and socioeconomic status is well characterised in the literature. However, there is an absence of evidence assessing socioeconomic status in enrolment to trials, which is what we aim to assess in this study. This is important not just for the generalisability of trial results but from an equity perspective to ensure that all individuals in the target population can engage with the trial results. In our limitation section, we have expanded on how these results may not be representative of other settings (page 17):

  "Both TASTE and VALIDATE investigated ~~acute cardiac interventions~~ point interventions delivered in acute cardiac settings. Additionally, healthcare in Sweden is predominantly tax-funded and freely available (44). ~~Between 48 and 61% of screened enrolled across the two trials, a proportion that may not be representative of other settings~~ Therefore, both the proportion of individuals who were enrolled (38,45–47) and the observed socioeconomic differences may not be representative of other countries or clinical contexts outside of acute cardiac interventions."

Reproducibility

- Analytical code is available via GitHub, but no data are available, so I cannot assess the reproducibility of the findings.
- Since the data were already pseudonymised, data sharing may still be feasible.

  Response - Swedish law and regulations do not permit us to share sensitive personal data, even when pseudonymised. However, researchers who meet the legal requirements including ethical approval can apply for data access from the relevant bodies.

- The code appears partially reproducible. Bootstrap and seeds are provided, which is ok, but there are inconsistencies such as handling of lopnr (removed and later reused). Please double check whether the workflow is fully reproducible. Alternatively please get an independent CODECHEK.

  Response - Lopnr is the main individual identifier, which is used to link datasets. We remove it in the script func_tableone from the dataset that generates the

comparison of standardised mean differences between enrolled and non enrolled individuals (also correspondingly in the script func_tableone_clin).

For the random forest model (func_randomforest_cv_10fold_hyper.R script), a bootstrap (resample of the data with replacement) of the data is taken. Within each bootstrap, any individuals who are in the testing data are removed from the training data set to prevent data leakage. Lopnr is then removed so that the random forest does not treat it as a predictive covariate.

Lopnr is additionally removed from the dataset which is used to set up the hyperparameter grid for the random forest model (within the training data set). This is to ensure that the correct number of variables are used in the random forest.

Analytic code is routinely checked by members of the team. The workflow uploaded to GitHub has been double checked and is fully reproducible.

Transparency

- Please provide links to study registration and/or study protocol, if available.

  Response - This study was not preregistered.

- Please provide a completed reporting checklist such as STROBE or TRIPOD.

  Response - We have attached the STROBE checklist.

- The coding workflow is scripted and annotated, which is appreciated.

  Response - Thank you.

Reviewer: 2

Dr. Mulugeta Birhanu, Monash Health
Comments to the Author:
Reviewer comments

The paper entitled “Differences in socioeconomic characteristics of individuals who are and are not enrolled in cardiovascular registry based randomised controlled trials” has the potential to contribute to the existing body of literature. This is a well written manuscript. However, substantial revision is required regarding the methods, interpretation of the findings, and discussion of the potential implications and limitations.

Major comments:

- The authors should provide further clarification and discuss regarding the reasons why eligible individuals did not participate in the TASTE and VALIDATE-SWEDEHEART trials. Is that because of restrictive eligibility criteria, reduce engagement, language barrier, concern or lack of trust in clinical trial, consent, or other reason? This information would help readers to better understand the reasons for the differences.

  Response - We have amended the text as follows to provide further detail on the non-enrolled population (page 16):

  "When data on non-enrolled individuals are available, a challenge arises due to the varying reasons for non-enrolment. In TASTE and VALIDATE-SWEDEHEART, many individuals in the non-enrolled group were ineligible due to not fulfilling a clinical criterion or lack of consent ~~(either due to declining or being unable to provide it),~~ while some were not asked to enrol or were excluded for reasons not listed in the trial protocols. Non-enrolment may therefore result from an individual's decision to decline participation, which may reflect broader levels of trust in and engagement with the healthcare system. It may also arise from an inability to provide informed consent, for example because of clinical condition or language barriers. Finally, non-enrolment may stem from differences in recruitment practices and trial engagement at the clinician, hospital, and regional levels. This may in part explain the marked variation in enrolment observed across regions of residence in this study. As levels of socioeconomic deprivation vary both between and within regions in Sweden (42), socioeconomic status alone is unlikely to explain this finding. It was not possible to identify otherwise clinically eligible individuals who were not asked to participate.

  ~~This~~ The varied reasons for non-enrolment raise~~s~~ the question of whether these individuals would qualify for the target population, where consent would not be an inclusion criterion. Here, given the reasons for exclusion, it's likely that a substantial portion of the non-enrolled group would be in the target population."

- Even though the authors preferred to analyse the Sweden data, there is still a variation in region of residence. The authors should provide further discussion of the potential reasons for these regional differences. For example, could these differences be explained by variation in the sociodemographic characteristics of the regions, differences in access to participating trial centres, or other contextual factors.

  Response - Thank you for bringing up this valuable point. Assessment of socioeconomic characteristics was limited to participants in Sweden for whom linked socioeconomic data were available. We have amended the discussion as follows (page 16):

  "Finally, non-enrolment may stem from differences in recruitment practices and trial engagement at the clinician, hospital, and regional levels. This may in part explain the marked variation in enrolment observed across regions of residence in this study. As

levels of socioeconomic deprivation vary both between and within regions in Sweden (42), socioeconomic status alone is unlikely to explain this finding."

- The author provided the SMD rather than P-values for comparison between groups. For the readers, providing clear explanation on the interpretation of the SMDs would essential.

  Response - We report SMD instead of p values to give readers an indication of the magnitude of the size of the differences. We have amended the text as follows (page 6):

  "Baseline socioeconomic characteristics of the enrolled and non-enrolled groups at the point of recruitment were compared using standardised mean differences. Standardised mean differences (SMD) quantify the magnitude of differences between groups. We considered SMD values exceeding 0.1 standard deviations to be indicative of meaningful differences."

- In the summary, the authors mentioned that as they found clear socioeconomic differences between individuals who were and were not enrolled in the trials but the definition for socioeconomic is broad and should be interpreted with caution.

  Response: We have amended the text as follow (page 17):

  "In summary, we found ~~clear socioeconomic~~ differences in multiple socio-economic domains between individuals who were and were not enrolled in two cardiovascular registry-based randomised trials."

- The authors also need to discuss the potential reasons of why women are less likely to be involved in trials than men? Is that because of eligibility, comorbidity, socioeconomic circumstances, caregiving responsibilities, health seeking behaviour, lack of transport, willingness, structural and social factors, and others? And the result also did not include information regarding the discrepancies between men and women.

  Response - We did not explicitly focus on why women are less likely to enrol in this manuscript as this is well characterised in the literature, however we have added further detail to provide a broader context (page 15):

  "In this study, both women and older individuals were less likely to be enrolled, which is consistent with previous evidence (34–37). This may reflect factors related to the trial (e.g., restrictive eligibility criteria), individual-level factors (e.g., engagement with and trust in the clinical trial process), and the broader healthcare environment (e.g., which patients are approached for enrolment) (34-36)."

- Statistical analysis: The variables included in the clinical covariates, socioeconomic characteristics, and both clinical and socioeconomic covariate models are not clearly specified in the manuscript. The authors should provide the detailed list of variables

included in each model and explain how these variables were selected. In particular, it would be helpful to clarify whether the variables were selected a priori based on clinical or theoretical considerations, based on previous literature, or using a data-driven approach.

Response: For the clinical covariate models, we used covariates which were available in the original trial publications and had less than 0.5% missing (full reasoning provided in the supplementary appendix). We have clarified in the text (page 7):

"A random forest was used to derive a predictive model for enrolment using the baseline clinical covariates available in the original trials... Due to the varying proportion of missing data between the enrolled and non-enrolled groups, only variables with less than 0.5% missing were included in the clinical model. Further ~~full~~ details are provided in the Supplementary Appendix (including Supplementary Tables 2-5). Clinical models included age, sex, previous PCI and previous coronary artery bypass grafting (TASTE only: warfarin use before PCI, heparin use before PCI, and thrombolysis before PCI, and VALIDATE-SWEDEHEART only: myocardial infarction type)."

We have clarified how the socioeconomic covariates were chosen (page 6):

"For this study, ~~additional~~ socioeconomic variables were obtained from LISA relating to migration, residential characteristics, family structure, education, occupation and income~~. (See~~) Supplementary Table 1 contains ~~for~~ the full list of included variables ~~list~~. These variables were chosen a priori using a theory-informed approach to capture a comprehensive range of socioeconomic status dimensions relevant to the study aim."

- I suggest the to provide the predictive model performance results in Table in the main manuscript as they did for sensitivity analysis in the online supplement.

  Response - We have amended this.

- The generalisability of the finding to other trial is quite limited and should be discussed more explicitly.

  Response – We have amended the text as follows (page 17):

  "Both TASTE and VALIDATE investigated ~~acute cardiac interventions~~ point interventions delivered in acute cardiac settings. Additionally, healthcare in Sweden is predominantly tax-funded and freely available (44). ~~Between 48 and 61% of screened enrolled across the two trials, a proportion that may not be representative of other settings~~ Therefore, both the proportion of individuals who enrolled (38,45–47) and the observed socioeconomic differences may not be representative of other countries or clinical contexts outside of acute cardiac interventions."

Minor comments

- There are a relatively large number of Tables in the manuscript. I suggest consolidating tables where possible to improve readability and reduce redundancy. For example, Supplementary Tables 4 and 5 could be combined into a single Table.

Response – We have combined Supplementary Tables 4 and 5.

- Result page 9 of 81 (line 16): I suggest removing the phrase "have no occupational status or" as it is confusing to readers.

Response - We have changed the phrase "have no occupational status" to "are retired"

General comments:

The authors provided an interesting and important area of research but the methods, result and discussion require major revision. The implication or the interpretation of the findings needs some caution.

VERSION 2 - REVIEW

**Reviewer**	**1**
**Name**	**Dewi, Ayu Putu Madri**
**Affiliation**	**Amsterdam UMC Location AMC, Epidemiology and Data Science**
**Date**	**09-Oct-2026**
**COI**	**NA**

Thank you for the revisions and detailed responses. I consider that the authors have addressed my concerns.

One minor suggestion remains regarding the second study aim. Please consider clarifying the term "additional collective value" to explain more explicitly what is being assessed, namely, whether socioeconomic variables provide additional discriminatory value beyond routinely collected clinical variables in predicting trial enrolment. Given the diversity of socioeconomic variables, it would be helpful to clarify this aim.

I have no further comments.

VERSION 2 - AUTHOR RESPONSE

Reviewer one:

Thank you for the revisions and detailed responses. I consider that the authors have addressed my concerns.

One minor suggestion remains regarding the second study aim. Please consider clarifying the term “additional collective value” to explain more explicitly what is being assessed, namely, whether socioeconomic variables provide additional discriminatory value beyond routinely collected clinical variables in predicting trial enrolment. Given the diversity of socioeconomic variables, it would be helpful to clarify this aim.

I have no further comments.

Response: We have amended the text as follows (page 5):

“Second, we aim to assess whether socioeconomic variables provide ~~the~~ additional discriminatory ~~collective~~ value ~~of socioeconomic variables~~ beyond routinely collected clinical data in predicting enrolment.”
