# Supplementary material for: Socioeconomic characteristics of individuals who were and were not enrolled in two cardiovascular registry-based randomised controlled trials in Sweden: a descriptive cross-sectional study
Source: BMJ Open. 2026 Nov 10;16(11):e121876. doi: 10.1136/bmjopen-2026-121876 (PMC13756278; doi:10.1136/bmjopen-2026-121876)
Supplement: Supplementary file 2 [file bmjopen-16-11-s001.docx]

Supplementary appendix

Contents

[Supplementary Table 1 – Covariate definitions	3](#_Toc241156130)

[Missing data in the clinical random forest predictive model for enrolment	5](#_Toc241156131)

[Supplementary Table 2: Baseline trial characteristics of individuals in Sweden who were and were not enrolled in the TASTE trial who are present in the study population for the socioeconomic analysis. NB continuous variables have been categorised to assess the proportion of missing data.	6](#_Toc241156132)

[Supplementary Table 3: Baseline trial characteristics of individuals who were and were not enrolled in the VALIDATE-SWEDEHEART trial who are present in the study population for the socioeconomic analysis. NB continuous variables have been categorised to assess the proportion of missing data.	8](#_Toc241156133)

[Supplementary Table 4: Number and percentage of missing data for the socioeconomic variables for individuals who were and were not enrolled in the TASTE and VALIDATE-SWEDEHEART trials.	10](#_Toc241156134)

[Model development and validation	12](#_Toc241156135)

[Supplementary Table 5: Baseline characteristics of individuals aged 65 years or older who were and were not enrolled in the TASTE trial	14](#_Toc241156136)

[Supplementary Table 6: Baseline characteristics of individuals aged under 65 years who were and were not enrolled in the TASTE trial	16](#_Toc241156137)

[Supplementary Table 7: Baseline characteristics of male individuals who were and were not enrolled in the TASTE trial	18](#_Toc241156138)

[Supplementary Table 8: Baseline characteristics of female individuals who were and were not enrolled in the TASTE trial	20](#_Toc241156139)

[Supplementary Table 9: Baseline characteristics of individuals aged 65 years or older who were and were not enrolled in the VALIDATE-SWEDEHEART trial	22](#_Toc241156140)

[Supplementary Table 10: Baseline characteristics of individuals aged under 65 years who were and were not enrolled in the VALIDATE-SWEDEHEART trial	24](#_Toc241156141)

[Supplementary Table 11: Baseline characteristics of male individuals who were and were not enrolled in the VALIDATE-SWEDEHEART trial	26](#_Toc241156142)

[Supplementary Table 12: Baseline characteristics of female individuals who were and were not enrolled in the VALIDATE-SWEDEHEART trial	28](#_Toc241156143)

[Supplementary Table 13: Sensitivity analysis for the random forest predictive model for enrolment using complete case analysis	30](#_Toc241156144)

Supplementary Table 1 – Covariate definitions

| **Covariate** |  | **Definition** | **Values** | **Form** |
| --- | --- | --- | --- | --- |
| **Age** |  | Age at trial recruitment (years) |  | Continuous |
| **Sex** |  |  | 1 = male, 2 = female | Categorical |
| **Country of birth** |  |  | 1 = Sweden, 2 = Other Nordic country, 3 = Europe excluding Nordic countries, 4 = North America, 5 = North and South America, 5 = Other (Africa, Asia, Soviet Union, Oceania) | Categorical |
| **Immigration in the 3 years prior** |  | Record of immigration to Sweden in the last 3 years prior | 0 = no, 1 = yes  If missing, assumed no. | Categorical |
| **Region of residence** |  | Recorded region of residence in last 3 years | 1 = Stockholm (Stockholm and Gotland), 2 = Uppsala (Uppsala, Södermanland, Värmland, Örebro, Västmanland, Kopparberg, Gävleborg), 3 = Linköping (Östergötland, Jönköping, Kalmar), 4 = Lund-Malmö (Kronoberg, Blekinge, Kristianstad, Malmöhus, Hylte, Halmstad, Laholm), 5 = Gothenburg (Göteborg & Bohus, Älvsborg, Skaraborg, Falkenberg, Varberg, Kungsbacka), 6 = Umeå (Västernorrland, Jämtland, Västerbotten, Norrbotten) | Categorical |
| **Degree of urbanisation** |  | Degree of urbanisation of region of residence | 1 = most urbanised, 2 = mid = urbanised, 3 = least urbanised |  |
| **Moved municipality in the last year** |  | Record of move to different municipality in the last year | 0 = no, 1 = yes | Categorical |
| **Divorce, separation, or loss of partner in last 3 years** |  | Record of divorce, separation or partner death in the last three years | 0 = no, 1 = yes | Categorical |
| **Family position** |  | Last recorded family or relationship status in the last 3 years | 1 = single, 2 = single parent, 3 = married, cohabiting or in a partnership | Categorical |
| **Education area** |  | Last recorded education subject in the last 3 years | 1 = general education, 2 = social sciences (teaching, humanities, art, social science, law and business) 3 = health and science (science, technology, agriculture, health and social care), 4 = Services | Categorical |
| **Number of years of education** |  | Last recorded number of years of education in the last 3 years | 1 = primary education ≤9 years, 2 = secondary education ≤3 years, 3 = tertiary education </> 3 years | Categorical |
| **Occupational status** |  | Most recent occupational status in last three years– NB for some data collection years, the recorded value relates to previous years as the most recent data available for that year | 1 = senior officials, managers and professionals 2 = small business owners and farmers, 3 = supervisors, technicians and skilled manual workers, 4 = other office employees, skilled workers in trade, services and care 5 = other manual worker, 6 = unemployed or retired status | Categorical |
| **At least one day of unemployment last 3 years** |  | At least one day recorded as unemployed in the last three years | 0 = no, 1 = yes | Categorical |
| **Individual yearly disposable income** |  | The mean yearly disposable income per hundred SEK by individual across the last 3 years |  | Quintile or Continuous |

**Missing data in the clinical random forest predictive model for enrolment**

The difference in the proportion of missing data for clinical trial covariates ranged from 0% to 13.0% between the enrolled and non-enrolled groups (see Supplementary Tables 2-3). It was not possible to determine the temporal order of baseline information being recorded relative to trial enrolment, meaning some data in the enrolled group may have been recorded after enrolment. To ensure that missing data was not influenced by enrolment status, only variables with no or little missing data (<0.5%) were included in the baseline clinical model. This 0.5% cut off was only applied to the clinical covariates due to the direct potential influence of enrolment status, which would not occur for socioeconomic data (the proportion of missing data can be seen in Supplementary Tables 4-5).

Supplementary Table 2: Baseline trial characteristics of individuals in Sweden who were and were not enrolled in the TASTE trial who are present in the study population for the socioeconomic analysis. NB continuous variables have been categorised to assess the proportion of missing data.

| Variable | Enrolled | Not enrolled | SMD | Missing |
| --- | --- | --- | --- | --- |
| N | 6841 | 4595 |  |  |
| Age — yr (mean ± SD) | 66.5 (11.6) | 68.8 (12.8) | 0.193 | 0 |
| Female sex — no. (%) | 1746 (25.5) | 1478 (32.2) | 0.147 | 0 |
| Body-mass index — mean ± SD | 27.1 (6.3) | 27.0 (8.8) | 0.016 | 4.3 |
| Body-mass index tertile — no. (%) |  |  | 0.208 | 4.3 |
| 1 | 2148 (31.4) | 1522 (33.1) |  |  |
| 2 | 2242 (32.8) | 1386 (30.2) |  |  |
| 3 | 2271 (33.2) | 1376 (29.9) |  |  |
| Missing | 180 (2.6) | 311 (6.8) |  |  |
| Diabetes — no. (%) |  |  | 0.226 | 1.4 |
| 0 | 5939 (86.8) | 3683 (80.2) |  |  |
| 1 | 870 (12.7) | 786 (17.1) |  |  |
| Missing | 32 (0.5) | 126 (2.7) |  |  |
| Current smoker — no. (%) |  |  | 0.307 | 10 |
| 0 | 2359 (34.5) | 1603 (34.9) |  |  |
| 1 | 1977 (28.9) | 1127 (24.5) |  |  |
| 2 | 2072 (30.3) | 1158 (25.2) |  |  |
| Missing | 433 (6.3) | 707 (15.4) |  |  |
| Hyperlipidemia — no. (%) |  |  | 0.279 | 4 |
| 0 | 5280 (77.2) | 3090 (67.2) |  |  |
| 1 | 1420 (20.8) | 1191 (25.9) |  |  |
| Missing | 141 (2.1) | 314 (6.8) |  |  |
| Hypertension — no. (%) |  |  | 0.275 | 3 |
| 0 | 3824 (55.9) | 2116 (46.1) |  |  |
| 1 | 2921 (42.7) | 2228 (48.5) |  |  |
| Missing | 96 (1.4) | 251 (5.5) |  |  |
| Previous myocardial infarction — no. (%) |  |  | 0.301 | 2.9 |
| 0 | 5955 (87.0) | 3530 (76.8) |  |  |
| 1 | 799 (11.7) | 819 (17.8) |  |  |
| Missing | 87 (1.3) | 246 (5.4) |  |  |
| Previous PCI — no. (%) |  |  | 0.094 | 0 |
| 0 | 6177 (90.3) | 4024 (87.6) |  |  |
| 1 | 664 (9.7) | 567 (12.3) |  |  |
| Missing | 0 (0.0) | 4 (0.1) |  |  |
| Previous CABG — no. (%) |  |  | 0.164 | 0.1 |
| 0 | 6699 (97.9) | 4360 (94.9) |  |  |
| 1 | 140 (2.0) | 230 (5.0) |  |  |
| Missing | 2 (0.0) | 5 (0.1) |  |  |
| Warfarin use before PCI — no. (%) |  |  | 0.075 | 0.2 |
| 0 | 6723 (98.3) | 4466 (97.2) |  |  |
| 1 | 111 (1.6) | 117 (2.5) |  |  |
| Missing | 7 (0.1) | 12 (0.3) |  |  |
| Heparin use before PCI — no. (%) |  |  | 0.152 | 0.2 |
| 0 | 4169 (60.9) | 3118 (67.9) |  |  |
| 1 | 2664 (38.9) | 1464 (31.9) |  |  |
| Missing | 8 (0.1) | 13 (0.3) |  |  |
| Thrombolysis before PCI — no. (%) |  |  | 0.042 | 0.2 |
| 0 | 6705 (98.0) | 4480 (97.5) |  |  |
| 1 | 128 (1.9) | 103 (2.2) |  |  |
| Missing | 8 (0.1) | 12 (0.3) |  |  |
| Time from ECG to PCI — median [IQR], min | 66.0 [47.0, 94.0] | 70.0 [49.0, 104.0] | 0.021 | 0.7 |
| Time from ECG to PCI tertile — no. (%) |  |  | 0.142 | 0.7 |
| 1 | 2440 (35.7) | 1503 (32.7) |  |  |
| 2 | 2278 (33.3) | 1398 (30.4) |  |  |
| 3 | 2093 (30.6) | 1639 (35.7) |  |  |
| Missing | 30 (0.4) | 55 (1.2) |  |  |
| Time from symptom onset to PCI — median [IQR], min | 183.0 [120.0, 323.0] | 200.0 [120.0, 400.0] | 0.021 | 19.8 |
| Time from symptom onset to PCI tertile — no. (%) |  |  | 0.339 | 19.8 |
| 1 | 2002 (29.3) | 1076 (23.4) |  |  |
| 2 | 2018 (29.5) | 1028 (22.4) |  |  |
| 3 | 1821 (26.6) | 1225 (26.7) |  |  |
| Missing | 1000 (14.6) | 1266 (27.6) |  |  |
| Killip class — no. (%) |  |  | 0.377 | 1.8 |
| 1 | 6414 (93.8) | 3793 (82.5) |  |  |
| 2 | 251 (3.7) | 350 (7.6) |  |  |
| 3 | 40 (0.6) | 99 (2.2) |  |  |
| 4 | 45 (0.7) | 238 (5.2) |  |  |
| Missing | 91 (1.3) | 115 (2.5) |  |  |

Supplementary Table 3: Baseline trial characteristics of individuals who were and were not enrolled in the VALIDATE-SWEDEHEART trial who are present in the study population for the socioeconomic analysis. NB continuous variables have been categorised to assess the proportion of missing data.

| Variable | Enrolled | Not enrolled | SMD | Missing |
| --- | --- | --- | --- | --- |
| N | 5947 | 6555 |  |  |
| STEMI — no. (%) | 2970 (49.9) | 4237 (64.6) | 0.3 | 0 |
| Female sex — no. (%) | 1567 (26.3) | 2000 (30.5) | 0.092 | 0 |
| Age — median [IQR], yr | 68.0 [60.0, 75.0] | 71.0 [62.0, 79.0] | 0.235 | 0 |
| Body-mass index — median [IQR] | 26.9 [24.5, 29.7] | 26.9 [24.2, 30.1] | 0.008 | 14 |
| Body-mass index tertile — no. (%) |  |  | 0.237 | 14 |
| 1 | 1718 (28.9) | 1865 (28.5) |  |  |
| 2 | 1891 (31.8) | 1693 (25.8) |  |  |
| 3 | 1743 (29.3) | 1839 (28.1) |  |  |
| Missing | 595 (10.0) | 1158 (17.7) |  |  |
| Low body weight — no. (%) |  |  | 0.133 | 0.9 |
| 0 | 5634 (94.7) | 6005 (91.6) |  |  |
| 1 | 287 (4.8) | 469 (7.2) |  |  |
| Missing | 26 (0.4) | 81 (1.2) |  |  |
| Current smoker — no. (%) |  |  | 0.206 | 4.7 |
| Non | 2347 (39.5) | 2441 (37.2) |  |  |
| Previous | 2037 (34.3) | 2314 (35.3) |  |  |
| Current | 1409 (23.7) | 1361 (20.8) |  |  |
| Missing | 154 (2.6) | 439 (6.7) |  |  |
| Diabetes — no. (%) |  |  | 0.222 | 0.5 |
| 0 | 4945 (83.2) | 4871 (74.3) |  |  |
| 1 | 987 (16.6) | 1631 (24.9) |  |  |
| Missing | 15 (0.3) | 53 (0.8) |  |  |
| Hypertension — no. (%) |  |  | 0.271 | 1.2 |
| 0 | 2847 (47.9) | 2349 (35.8) |  |  |
| 1 | 3072 (51.7) | 4080 (62.2) |  |  |
| Missing | 28 (0.5) | 126 (1.9) |  |  |
| Hyperlipidemia — no. (%) |  |  | 0.302 | 1.5 |
| 0 | 4035 (67.8) | 3520 (53.7) |  |  |
| 1 | 1866 (31.4) | 2893 (44.1) |  |  |
| Missing | 46 (0.8) | 142 (2.2) |  |  |
| Previous myocardial infarction — no. (%) |  |  | 0.305 | 1.9 |
| 0 | 4906 (82.5) | 4564 (69.6) |  |  |
| 1 | 959 (16.1) | 1831 (27.9) |  |  |
| Missing | 82 (1.4) | 160 (2.4) |  |  |
| Previous PCI — no. (%) |  |  | 0.19 | 0 |
| 0 | 5076 (85.4) | 5117 (78.1) |  |  |
| 1 | 871 (14.6) | 1436 (21.9) |  |  |
| Missing | 0 (0.0) | 2 (0.0) |  |  |
| Previous CABG — no. (%) |  |  | 0.199 | 0 |
| 0 | 5660 (95.2) | 5900 (90.0) |  |  |
| 1 | 287 (4.8) | 653 (10.0) |  |  |
| Missing | 0 (0.0) | 2 (0.0) |  |  |
| Previous stroke — no. (%) |  |  | 0.167 | 2.9 |
| 0 | 5583 (93.9) | 5851 (89.3) |  |  |
| 1 | 234 (3.9) | 467 (7.1) |  |  |
| Missing | 130 (2.2) | 237 (3.6) |  |  |
| CPR before hospital arrival — no. (%) |  |  | 0.116 | 4.1 |
| 0 | 5722 (96.2) | 6141 (93.7) |  |  |
| 1 | 46 (0.8) | 83 (1.3) |  |  |
| Missing | 179 (3.0) | 331 (5.0) |  |  |
| Killip class — no. (%) |  |  | 0.388 | 12.7 |
| 1 | 5294 (89.0) | 4907 (74.9) |  |  |
| 2 | 138 (2.3) | 260 (4.0) |  |  |
| 3 | 32 (0.5) | 92 (1.4) |  |  |
| 4 | 23 (0.4) | 167 (2.5) |  |  |
| Missing | 460 (7.7) | 1129 (17.2) |  |  |

Supplementary Table 4: Number and percentage of missing data for the socioeconomic variables for individuals who were and were not enrolled in the TASTE and VALIDATE-SWEDEHEART trials.

|  | **TASTE** | | **VALIDATE** | |
| --- | --- | --- | --- | --- |
| **Variable** | **Enrolled (6841)** | **Not enrolled (4595)** | **Enrolled (5947)** | **Not enrolled (6555)** |
| Age | 0 | 0 | 0 | 0 |
| Female | 0 | 0 | 0 | 0 |
| Country of birth | 0 | 0 | 0 | 0 |
| Immigration in the 3 years prior | 0 | 0 | 0 | 0 |
| Region of residence | 814 (11.9) | 613 (13.3) | 656 (11.0) | 651 (9.9) |
| Degree of urbanisation | 814 (11.9) | 613 (13.3) | 656 (11.0) | 651 (9.9) |
| Moved municipality in last year | 818 (12.0) | 616 (13.4) | 656 (11.0) | 651 (9.9) |
| Family position | 17 (0.2) | 9 (0.2) | 9 (0.2) | 30 (0.5) |
| Divorce, separation, or loss of partner in last 3 years | 17 (0.2) | 9 (0.2) | 9 (0.2) | 30 (0.5) |
| Education area | 144 (2.1) | 186 (4.0) | 125 (2.1) | 257 (3.9) |
| Education length | 70 (1.0) | 118 (2.6) | 39 (0.7) | 151 (2.3) |
| Occupational status | 31 (0.5) | 19 (0.4) | 46 (0.8) | 129 (2.0) |
| At least one day of unemployment last 3 years | 17 (0.2) | 9 (0.2) | 9 (0.2) | 30 (0.5) |
| Individual disposable income per hundred SEK | 17 (0.2) | 9 (0.2) | 9 (0.2) | 30 (0.5) |

**Model development and validation**

To estimate the area under the receiver operating characteristic curve (AUC) for the random forest model predicting enrolment, we used the *ranger* package in R with parallel processing across multiple central processing units to reduce computation time. Bootstrapping was used to estimate the point estimate and derive corresponding 95% confidence intervals. The following steps were taken:

For each bootstrap iteration, a bootstrap sample was generated by randomly sampling observations from the original dataset with replacement until the sample size matched that of the original dataset.

Within each bootstrap, the data were formatted to address missing values (either to include a missing indicator, or for the sensitivity analysis, a complete case analysis) and then 10-fold cross-validation was applied. The dataset was accordingly partitioned into 10 folds, with nine folds used for training and one for testing.

To prevent data leakage, any observations appearing in both sets were removed from the training set, after which individual identifiers were removed from both training and testing sets.

Within the training set, 80% of observations were used for hyperparameter tuning of the random forest model (number of trees, minimum node size, and number of nodes).

The hyperparameter combination that maximised the AUC in the remaining 20% of the training data was selected.

The model was then retrained on the full training dataset, before being validated on the remaining held-out testing fold, where it was used to generate predicted probabilities of enrolment.

This procedure was repeated ten times such that each fold served once as the test set.

Predicted probabilities from all folds were aggregated and compared with observed enrolment status to compute the bootstrap-specific AUC.

AUCs across all bootstrap iterations were then summarised by their mean, and the 2.5th and 97.5th percentiles were used to construct the 95% confidence interval.

Supplementary Table 5: Baseline characteristics of individuals aged 65 years or older who were and were not enrolled in the TASTE trial

| Variable | Enrolled | Not enrolled | Missing | SMD |
| --- | --- | --- | --- | --- |
| N | 3893 | 2924 |  |  |
| *Demographics and migration* |  |  |  |  |
| Age (mean (SD)) | 74.5 (7.0) | 76.6 (7.7) | 0 | 0.286 |
| Female | 1220 (31.3) | 1128 (38.6) | 0 | 0.152 |
| Country of birth |  |  | 0 | 0.161 |
| Sweden | 3482 (89.4) | 2492 (85.2) |  |  |
| Other Nordic countries | 206 (5.3) | 163 (5.6) |  |  |
| Europe excluding Nordics | 158 (4.1) | 192 (6.6) |  |  |
| North and South America | 10 (0.3) | 9 (0.3) |  |  |
| Other | 37 (1.0) | 68 (2.3) |  |  |
| Immigration in the 3 years prior | 5 (0.1) | 12 (0.4) | 0 | 0.054 |
| Region of residence |  |  | 11.9 | 0.263 |
| Stockholm | 396 (11.5) | 412 (16.1) |  |  |
| Uppsala | 972 (28.2) | 625 (24.4) |  |  |
| Linkoping | 370 (10.7) | 420 (16.4) |  |  |
| Lund-Malmo | 656 (19.0) | 475 (18.6) |  |  |
| Gothenburg | 780 (22.6) | 409 (16.0) |  |  |
| Umeå | 270 (7.8) | 218 (8.5) |  |  |
| Degree of urbanisation |  |  | 11.9 | 0.014 |
| Most urbanised | 1011 (29.4) | 768 (30.0) |  |  |
| Mid urbanised | 1552 (45.1) | 1144 (44.7) |  |  |
| Least urbanised | 881 (25.6) | 647 (25.3) |  |  |
| Moved municipality in last year | 40 (1.2) | 35 (1.4) | 12 | 0.018 |
| *Family structure* |  |  |  |  |
| Family position |  |  | 0.1 | 0.168 |
| Single | 1507 (38.8) | 1357 (46.4) |  |  |
| Single parent | 50 (1.3) | 54 (1.8) |  |  |
| Married, cohabiting, in a partnership | 2329 (59.9) | 1511 (51.7) |  |  |
| Divorce, separation, or loss of partner in last 3 years | 97 (2.5) | 88 (3.0) | 0.1 | 0.032 |
| *Education* |  |  |  |  |
| Education area |  |  | 2.5 | 0.122 |
| General education | 1799 (47.0) | 1464 (51.9) |  |  |
| Social sciences | 616 (16.1) | 407 (14.4) |  |  |
| Science, technology, agriculture, health and social care | 1183 (30.9) | 749 (26.6) |  |  |
| Services | 230 (6.0) | 199 (7.1) |  |  |
| Education length |  |  | 1.9 | 0.099 |
| Primary education ≤9yrs | 1744 (45.3) | 1420 (50.0) |  |  |
| Secondary education ≤3yrs | 1478 (38.4) | 1026 (36.1) |  |  |
| Tertiary education </>3yrs | 626 (16.3) | 394 (13.9) |  |  |
| *Employment and income* |  |  |  |  |
| Occupational status |  |  | 0.2 | 0.093 |
| Senior Officials, managers and professionals | 128 (3.3) | 63 (2.2) |  |  |
| Small business owners and farmers | 129 (3.3) | 89 (3.0) |  |  |
| Supervisors, technicians and skilled manual workers | 38 (1.0) | 28 (1.0) |  |  |
| Office employees and skilled workers in trade, services, and care | 52 (1.3) | 31 (1.1) |  |  |
| Other manual worker | 54 (1.4) | 25 (0.9) |  |  |
| Retired or unemployed | 3483 (89.7) | 2683 (91.9) |  |  |
| At least one day of unemployment last 3 years | 75 (1.9) | 29 (1.0) | 0.1 | 0.078 |
| Individual disposable income per hundred SEK | 1515.5 [1235.2, 2068.3] | 1381.8 [1156.0, 1816.6] | 0.1 | 0.108 |
| Individual disposable income per hundred SEK by quintile |  |  | 0.1 | 0.234 |
| 1 | 786 (20.2) | 799 (27.3) |  |  |
| 2 | 995 (25.6) | 856 (29.3) |  |  |
| 3 | 942 (24.2) | 622 (21.3) |  |  |
| 4 | 594 (15.3) | 320 (11.0) |  |  |
| 5 | 569 (14.6) | 325 (11.1) |  |  |

Supplementary Table 6: Baseline characteristics of individuals aged under 65 years who were and were not enrolled in the TASTE trial

| Variable | Enrolled | Not enrolled | Missing | SMD |
| --- | --- | --- | --- | --- |
| N | 2948 | 1671 |  |  |
| *Demographics and migration* |  |  |  |  |
| Age (mean (SD)) | 55.8 (6.7) | 55.1 (7.2) | 0 | 0.096 |
| Female | 526 (17.8) | 350 (20.9) | 0 | 0.079 |
| Country of birth |  |  | 0 | 0.268 |
| Sweden | 2412 (81.8) | 1205 (72.1) |  |  |
| Other Nordic countries | 142 (4.8) | 80 (4.8) |  |  |
| Europe excluding Nordics | 207 (7.0) | 191 (11.4) |  |  |
| North and South America | 28 (0.9) | 13 (0.8) |  |  |
| Other | 159 (5.4) | 182 (10.9) |  |  |
| Immigration in the 3 years prior | 25 (0.8) | 35 (2.1) | 0 | 0.104 |
| Region of residence |  |  | 13.3 | 0.329 |
| Stockholm | 373 (14.4) | 295 (20.7) |  |  |
| Uppsala | 705 (27.3) | 313 (22.0) |  |  |
| Linkoping | 230 (8.9) | 230 (16.2) |  |  |
| Lund-Malmo | 511 (19.8) | 285 (20.0) |  |  |
| Gothenburg | 519 (20.1) | 211 (14.8) |  |  |
| Umeå | 245 (9.5) | 89 (6.3) |  |  |
| Degree of urbanisation |  |  | 13.3 | 0.063 |
| Most urbanised | 867 (33.6) | 512 (36.0) |  |  |
| Mid urbanised | 1098 (42.5) | 603 (42.4) |  |  |
| Least urbanised | 618 (23.9) | 308 (21.6) |  |  |
| Moved municipality in last year | 55 (2.1) | 41 (2.9) | 13.4 | 0.048 |
| *Family structure* |  |  |  |  |
| Family position |  |  | 0.4 | 0.028 |
| Single | 1141 (38.8) | 631 (37.9) |  |  |
| Single parent | 145 (4.9) | 91 (5.5) |  |  |
| Married, cohabiting, in a partnership | 1652 (56.2) | 942 (56.6) |  |  |
| Divorce, separation, or loss of partner in last 3 years | 48 (1.6) | 30 (1.8) | 0.4 | 0.013 |
| *Education* |  |  |  |  |
| Education area |  |  | 3.5 | 0.081 |
| General education | 902 (31.4) | 559 (35.2) |  |  |
| Social sciences | 495 (17.3) | 269 (16.9) |  |  |
| Science, technology, agriculture, health and social care | 1302 (45.4) | 672 (42.3) |  |  |
| Services | 170 (5.9) | 90 (5.7) |  |  |
| Education length |  |  | 1.3 | 0.087 |
| Primary education ≤9yrs | 768 (26.3) | 475 (29.0) |  |  |
| Secondary education ≤3yrs | 1526 (52.2) | 784 (47.9) |  |  |
| Tertiary education </>3yrs | 629 (21.5) | 378 (23.1) |  |  |
| *Employment and income* |  |  |  |  |
| Occupational status |  |  | 0.8 | 0.21 |
| Senior Officials, managers and professionals | 556 (19.0) | 311 (18.8) |  |  |
| Small business owners and farmers | 234 (8.0) | 102 (6.2) |  |  |
| Supervisors, technicians and skilled manual workers | 315 (10.8) | 122 (7.4) |  |  |
| Office employees and skilled workers in trade, services, and care | 501 (17.1) | 241 (14.5) |  |  |
| Other manual worker | 512 (17.5) | 285 (17.2) |  |  |
| Retired or unemployed | 808 (27.6) | 596 (36.0) |  |  |
| At least one day of unemployment last 3 years | 493 (16.8) | 306 (18.4) | 0.4 | 0.042 |
| Individual disposable income per hundred SEK | 2224.0 [1581.8, 2852.2] | 2028.0 [1291.2, 2720.8] | 0.4 | 0.158 |
| Individual disposable income per hundred SEK by quintile |  |  | 0.4 | 0.243 |
| 1 | 361 (12.3) | 336 (20.2) |  |  |
| 2 | 257 (8.7) | 174 (10.5) |  |  |
| 3 | 456 (15.5) | 262 (15.7) |  |  |
| 4 | 926 (31.5) | 442 (26.6) |  |  |
| 5 | 938 (31.9) | 450 (27.0) |  |  |

Supplementary Table 7: Baseline characteristics of male individuals who were and were not enrolled in the TASTE trial

| Variable | Enrolled | Not enrolled | Missing | SMD |
| --- | --- | --- | --- | --- |
| N | 5095 | 3117 |  |  |
| *Demographics and migration* |  |  |  |  |
| Age (mean (SD)) | 65.1 (11.2) | 66.7 (12.4) | 0 | 0.137 |
| Male | 5095 (100.0) | 3117 (100.0) | 0 | <0.001 |
| Country of birth |  |  | 0 | 0.199 |
| Sweden | 4360 (85.6) | 2465 (79.1) |  |  |
| Other Nordic countries | 227 (4.5) | 145 (4.7) |  |  |
| Europe excluding Nordics | 297 (5.8) | 283 (9.1) |  |  |
| North and South America | 33 (0.6) | 17 (0.5) |  |  |
| Other | 178 (3.5) | 207 (6.6) |  |  |
| Immigration in the 3 years prior | 28 (0.5) | 38 (1.2) | 0 | 0.072 |
| Region of residence |  |  | 12.6 | 0.299 |
| Stockholm | 598 (13.3) | 513 (19.1) |  |  |
| Uppsala | 1252 (27.9) | 603 (22.4) |  |  |
| Linkoping | 439 (9.8) | 441 (16.4) |  |  |
| Lund-Malmo | 849 (18.9) | 522 (19.4) |  |  |
| Gothenburg | 939 (20.9) | 410 (15.3) |  |  |
| Umeå | 410 (9.1) | 199 (7.4) |  |  |
| Degree of urbanisation |  |  | 12.6 | 0.030 |
| Most urbanised | 1418 (31.6) | 869 (32.3) |  |  |
| Mid urbanised | 1946 (43.4) | 1180 (43.9) |  |  |
| Least urbanised | 1123 (25.0) | 639 (23.8) |  |  |
| Moved municipality in last year | 70 (1.6) | 57 (2.1) | 12.7 | 0.042 |
| *Family structure* |  |  |  |  |
| Family position |  |  | 0.3 | 0.029 |
| Single | 1771 (34.9) | 1125 (36.2) |  |  |
| Single parent | 118 (2.3) | 74 (2.4) |  |  |
| Married, cohabiting, in a partnership | 3192 (62.8) | 1909 (61.4) |  |  |
| Divorce, separation, or loss of partner in last 3 years | 96 (1.9) | 61 (2.0) | 0.3 | 0.005 |
| *Education* |  |  |  |  |
| Education area |  |  | 2.9 | 0.097 |
| General education | 1931 (38.8) | 1292 (43.2) |  |  |
| Social sciences | 728 (14.6) | 412 (13.8) |  |  |
| Science, technology, agriculture, health and social care | 2032 (40.8) | 1105 (36.9) |  |  |
| Services | 289 (5.8) | 185 (6.2) |  |  |
| Education length |  |  | 1.5 | 0.094 |
| Primary education ≤9yrs | 1786 (35.4) | 1209 (39.7) |  |  |
| Secondary education ≤3yrs | 2278 (45.2) | 1250 (41.0) |  |  |
| Tertiary education </>3yrs | 980 (19.4) | 588 (19.3) |  |  |
| *Employment and income* |  |  |  |  |
| Occupational status |  |  | 0.5 | 0.162 |
| Senior Officials, managers and professionals | 605 (11.9) | 314 (10.1) |  |  |
| Small business owners and farmers | 339 (6.7) | 169 (5.5) |  |  |
| Supervisors, technicians and skilled manual workers | 350 (6.9) | 146 (4.7) |  |  |
| Office employees and skilled workers in trade, services, and care | 317 (6.3) | 166 (5.4) |  |  |
| Other manual worker | 519 (10.2) | 275 (8.9) |  |  |
| Retired or unemployed | 2938 (58.0) | 2029 (65.5) |  |  |
| At least one day of unemployment last 3 years | 490 (9.6) | 283 (9.1) | 0.3 | 0.018 |
| Individual disposable income per hundred SEK | 1960.0 [1411.0, 2705.0] | 1709.0 [1286.0, 2505.2] | 0.3 | 0.122 |
| Individual disposable income per hundred SEK by quintile |  |  | 0.3 | 0.228 |
| 1 | 616 (12.1) | 561 (18.1) |  |  |
| 2 | 792 (15.6) | 599 (19.3) |  |  |
| 3 | 1069 (21.0) | 656 (21.1) |  |  |
| 4 | 1244 (24.5) | 621 (20.0) |  |  |
| 5 | 1360 (26.8) | 671 (21.6) |  |  |

Supplementary Table 8: Baseline characteristics of female individuals who were and were not enrolled in the TASTE trial

| Variable | Enrolled | Not enrolled | Missing | SMD |
| --- | --- | --- | --- | --- |
| N | 1746 | 1478 |  |  |
| *Demographics and migration* |  |  |  |  |
| Age (mean (SD)) | 70.4 (11.7) | 73.2 (12.5) | 0 | 0.231 |
| Female | 1746 (100.0) | 1478 (100.0) | 0 | <0.001 |
| Country of birth |  |  | 0 | 0.190 |
| Sweden | 1534 (87.9) | 1232 (83.4) |  |  |
| Other Nordic countries | 121 (6.9) | 98 (6.6) |  |  |
| Europe excluding Nordics | 68 (3.9) | 100 (6.8) |  |  |
| North and South America | 5 (0.3) | 5 (0.3) |  |  |
| Other | 18 (1.0) | 43 (2.9) |  |  |
| Immigration in the 3 years prior | 2 (0.1) | 9 (0.6) | 0 | 0.082 |
| Region of residence |  |  | 12.1 | 0.264 |
| Stockholm | 171 (11.1) | 194 (15.0) |  |  |
| Uppsala | 425 (27.6) | 335 (25.9) |  |  |
| Linkoping | 161 (10.5) | 209 (16.2) |  |  |
| Lund-Malmo | 318 (20.6) | 238 (18.4) |  |  |
| Gothenburg | 360 (23.4) | 210 (16.2) |  |  |
| Umeå | 105 (6.8) | 108 (8.3) |  |  |
| Degree of urbanisation |  |  | 12.1 | 0.044 |
| Most urbanised | 460 (29.9) | 411 (31.8) |  |  |
| Mid urbanised | 704 (45.7) | 567 (43.8) |  |  |
| Least urbanised | 376 (24.4) | 316 (24.4) |  |  |
| Moved municipality in last year | 25 (1.6) | 19 (1.5) | 12.1 | 0.013 |
| *Family structure* |  |  |  |  |
| Family position |  |  | 0.1 | 0.173 |
| Single | 877 (50.3) | 863 (58.4) |  |  |
| Single parent | 77 (4.4) | 71 (4.8) |  |  |
| Married, cohabiting, in a partnership | 789 (45.3) | 544 (36.8) |  |  |
| Divorce, separation, or loss of partner in last 3 years | 49 (2.8) | 57 (3.9) | 0.1 | 0.058 |
| *Education* |  |  |  |  |
| Education area |  |  | 2.9 | 0.155 |
| General education | 770 (44.8) | 731 (51.7) |  |  |
| Social sciences | 383 (22.3) | 264 (18.7) |  |  |
| Science, technology, agriculture, health and social care | 453 (26.4) | 316 (22.3) |  |  |
| Services | 111 (6.5) | 104 (7.3) |  |  |
| Education length |  |  | 2.1 | 0.128 |
| Primary education ≤9yrs | 726 (42.0) | 686 (48.0) |  |  |
| Secondary education ≤3yrs | 726 (42.0) | 560 (39.2) |  |  |
| Tertiary education </>3yrs | 275 (15.9) | 184 (12.9) |  |  |
| *Employment and income* |  |  |  |  |
| Occupational status |  |  | 0.2 | 0.217 |
| Senior Officials, managers and professionals | 79 (4.5) | 60 (4.1) |  |  |
| Small business owners and farmers | 24 (1.4) | 22 (1.5) |  |  |
| Supervisors, technicians and skilled manual workers | 3 (0.2) | 4 (0.3) |  |  |
| Office employees and skilled workers in trade, services, and care | 236 (13.5) | 106 (7.2) |  |  |
| Other manual worker | 47 (2.7) | 35 (2.4) |  |  |
| Retired or unemployed | 1353 (77.7) | 1250 (84.6) |  |  |
| At least one day of unemployment last 3 years | 78 (4.5) | 52 (3.5) | 0.1 | 0.049 |
| Individual disposable income per hundred SEK | 1360.0 [1102.7, 1901.0] | 1276.0 [1042.2, 1634.0] | 0.1 | 0.155 |
| Individual disposable income per hundred SEK by quintile |  |  | 0.1 | 0.254 |
| 1 | 531 (30.5) | 574 (38.8) |  |  |
| 2 | 460 (26.4) | 431 (29.2) |  |  |
| 3 | 329 (18.9) | 228 (15.4) |  |  |
| 4 | 276 (15.8) | 141 (9.5) |  |  |
| 5 | 147 (8.4) | 104 (7.0) |  |  |

Supplementary Table 9: Baseline characteristics of individuals aged 65 years or older who were and were not enrolled in the VALIDATE-SWEDEHEART trial

| Variable | Enrolled | Not enrolled | Missing | SMD |
| --- | --- | --- | --- | --- |
| N | 3630 | 4528 |  |  |
| *Demographics and migration* |  |  |  |  |
| Age (mean (SD)) | 74.3 (6.5) | 76.4 (7.2) | 0 | 0.307 |
| Female | 1139 (31.4) | 1596 (35.2) | 0 | 0.082 |
| Country of birth |  |  | 0 | 0.246 |
| Sweden | 3265 (89.9) | 3735 (82.5) |  |  |
| Other Nordic countries | 180 (5.0) | 291 (6.4) |  |  |
| Europe excluding Nordics | 143 (3.9) | 329 (7.3) |  |  |
| North and South America | 10 (0.3) | 23 (0.5) |  |  |
| Other | 32 (0.9) | 150 (3.3) |  |  |
| Immigration in the 3 years prior | 12 (0.3) | 28 (0.6) | 0 | 0.042 |
| Region of residence |  |  | 10.3 | 0.373 |
| Stockholm | 384 (12.0) | 902 (22.0) |  |  |
| Uppsala | 945 (29.4) | 897 (21.8) |  |  |
| Linkoping | 130 (4.0) | 299 (7.3) |  |  |
| Lund-Malmo | 703 (21.9) | 777 (18.9) |  |  |
| Gothenburg | 733 (22.8) | 685 (16.7) |  |  |
| Umeå | 315 (9.8) | 548 (13.3) |  |  |
| Degree of urbanisation |  |  | 10.3 | 0.017 |
| Most urbanised | 1029 (32.1) | 1333 (32.4) |  |  |
| Mid urbanised | 1422 (44.3) | 1785 (43.5) |  |  |
| Least urbanised | 759 (23.6) | 990 (24.1) |  |  |
| Moved municipality in last year | 53 (1.7) | 52 (1.3) | 10.3 | 0.032 |
| *Family structure* |  |  |  |  |
| Family position |  |  | 0.2 | 0.109 |
| Single | 1469 (40.5) | 2045 (45.3) |  |  |
| Single parent | 44 (1.2) | 78 (1.7) |  |  |
| Married, cohabiting, in a partnership | 2111 (58.3) | 2396 (53.0) |  |  |
| Divorce, separation, or loss of partner in last 3 years | 85 (2.3) | 142 (3.1) | 0.2 | 0.049 |
| *Education* |  |  |  |  |
| Education area |  |  | 2.6 | 0.104 |
| General education | 1434 (40.2) | 1984 (45.3) |  |  |
| Social sciences | 677 (19.0) | 769 (17.6) |  |  |
| Science, technology, agriculture, health and social care | 1218 (34.2) | 1358 (31.0) |  |  |
| Services | 237 (6.6) | 267 (6.1) |  |  |
| Education length |  |  | 1.5 | 0.118 |
| Primary education ≤9yrs | 1360 (37.7) | 1897 (42.8) |  |  |
| Secondary education ≤3yrs | 1487 (41.3) | 1770 (39.9) |  |  |
| Tertiary education </>3yrs | 756 (21.0) | 766 (17.3) |  |  |
| *Employment and income* |  |  |  |  |
| Occupational status |  |  | 0.5 | 0.174 |
| Senior Officials, managers and professionals | 220 (6.1) | 169 (3.8) |  |  |
| Small business owners and farmers | 218 (6.0) | 179 (4.0) |  |  |
| Supervisors, technicians and skilled manual workers | 53 (1.5) | 47 (1.0) |  |  |
| Office employees and skilled workers in trade, services, and care | 96 (2.7) | 97 (2.2) |  |  |
| Other manual worker | 83 (2.3) | 65 (1.4) |  |  |
| Retired or unemployed | 2946 (81.5) | 3942 (87.6) |  |  |
| At least one day of unemployment last 3 years | 54 (1.5) | 62 (1.4) | 0.2 | 0.01 |
| Individual disposable income per hundred SEK | 1706.0 [1396.7, 2522.7] | 1569.3 [1313.2, 2110.2] | 0.2 | 0.039 |
| Individual disposable income per hundred SEK by quintile |  |  | 0.2 | 0.217 |
| 1 | 650 (17.9) | 1064 (23.5) |  |  |
| 2 | 872 (24.1) | 1247 (27.6) |  |  |
| 3 | 861 (23.8) | 1061 (23.5) |  |  |
| 4 | 615 (17.0) | 573 (12.7) |  |  |
| 5 | 626 (17.3) | 574 (12.7) |  |  |

Supplementary Table 10: Baseline characteristics of individuals aged under 65 years who were and were not enrolled in the VALIDATE-SWEDEHEART trial

| Variable | Enrolled | Not enrolled | Missing | SMD |
| --- | --- | --- | --- | --- |
| N | 2317 | 2027 |  |  |
| *Demographics and migration* |  |  |  |  |
| Age (mean (SD)) | 56.0 (6.3) | 55.3 (7.0) | 0 | 0.103 |
| Female | 428 (18.5) | 404 (19.9) | 0 | 0.037 |
| Country of birth |  |  | 0 | 0.472 |
| Sweden | 1921 (82.9) | 1292 (63.7) |  |  |
| Other Nordic countries | 96 (4.1) | 99 (4.9) |  |  |
| Europe excluding Nordics | 147 (6.3) | 258 (12.7) |  |  |
| North and South America | 16 (0.7) | 24 (1.2) |  |  |
| Other | 137 (5.9) | 354 (17.5) |  |  |
| Immigration in the 3 years prior | 14 (0.6) | 90 (4.4) | 0 | 0.246 |
| Region of residence |  |  | 10.8 | 0.37 |
| Stockholm | 312 (15.0) | 469 (26.1) |  |  |
| Uppsala | 542 (26.0) | 354 (19.7) |  |  |
| Linkoping | 116 (5.6) | 144 (8.0) |  |  |
| Lund-Malmo | 447 (21.5) | 386 (21.5) |  |  |
| Gothenburg | 480 (23.1) | 254 (14.1) |  |  |
| Umeå | 184 (8.8) | 189 (10.5) |  |  |
| Degree of urbanisation |  |  | 10.8 | 0.071 |
| Most urbanised | 733 (35.2) | 684 (38.1) |  |  |
| Mid urbanised | 874 (42.0) | 747 (41.6) |  |  |
| Least urbanised | 474 (22.8) | 365 (20.3) |  |  |
| Moved municipality in last year | 54 (2.6) | 54 (3.0) | 10.8 | 0.025 |
| *Family structure* |  |  |  |  |
| Family position |  |  | 0.6 | 0.04 |
| Single | 875 (37.8) | 767 (38.2) |  |  |
| Single parent | 131 (5.7) | 131 (6.5) |  |  |
| Married, cohabiting, in a partnership | 1308 (56.5) | 1108 (55.2) |  |  |
| Divorce, separation, or loss of partner in last 3 years | 37 (1.6) | 46 (2.3) | 0.6 | 0.05 |
| *Education* |  |  |  |  |
| Education area |  |  | 3.9 | 0.144 |
| General education | 607 (26.9) | 628 (32.7) |  |  |
| Social sciences | 418 (18.5) | 312 (16.2) |  |  |
| Science, technology, agriculture, health and social care | 1096 (48.6) | 844 (44.0) |  |  |
| Services | 135 (6.0) | 136 (7.1) |  |  |
| Education length |  |  | 1.6 | 0.138 |
| Primary education ≤9yrs | 476 (20.7) | 513 (26.0) |  |  |
| Secondary education ≤3yrs | 1275 (55.3) | 976 (49.5) |  |  |
| Tertiary education </>3yrs | 554 (24.0) | 482 (24.5) |  |  |
| *Employment and income* |  |  |  |  |
| Occupational status |  |  | 3 | 0.337 |
| Senior Officials, managers and professionals | 521 (22.8) | 338 (17.5) |  |  |
| Small business owners and farmers | 185 (8.1) | 156 (8.1) |  |  |
| Supervisors, technicians and skilled manual workers | 247 (10.8) | 150 (7.8) |  |  |
| Office employees and skilled workers in trade, services, and care | 445 (19.5) | 280 (14.5) |  |  |
| Other manual worker | 412 (18.0) | 329 (17.1) |  |  |
| Retired or unemployed | 475 (20.8) | 674 (35.0) |  |  |
| At least one day of unemployment last 3 years | 389 (16.8) | 437 (21.8) | 0.6 | 0.126 |
| Individual disposable income per hundred SEK | 2550.0 [1788.9, 3311.9] | 2151.3 [1339.7, 3021.6] | 0.6 | 0.115 |
| Individual disposable income per hundred SEK by quintile |  |  | 0.6 | 0.341 |
| 1 | 314 (13.6) | 465 (23.2) |  |  |
| 2 | 166 (7.2) | 208 (10.4) |  |  |
| 3 | 271 (11.7) | 300 (15.0) |  |  |
| 4 | 783 (33.8) | 521 (26.0) |  |  |
| 5 | 780 (33.7) | 512 (25.5) |  |  |

Supplementary Table 11: Baseline characteristics of male individuals who were and were not enrolled in the VALIDATE-SWEDEHEART trial

| Variable | Enrolled | Not enrolled | Missing | SMD |
| --- | --- | --- | --- | --- |
| N | 4380 | 4555 |  |  |
| *Demographics and migration* |  |  |  |  |
| Age (mean (SD)) | 65.8 (10.7) | 68.2 (12.0) | 0 | 0.208 |
| Male | 4380 (100.0) | 4555 (100.0) | 0 | <0.001 |
| Country of birth |  |  | 0 | 0.307 |
| Sweden | 3804 (86.8) | 3456 (75.9) |  |  |
| Other Nordic countries | 180 (4.1) | 226 (5.0) |  |  |
| Europe excluding Nordics | 225 (5.1) | 423 (9.3) |  |  |
| North and South America | 19 (0.4) | 41 (0.9) |  |  |
| Other | 152 (3.5) | 409 (9.0) |  |  |
| Immigration in the 3 years prior | 21 (0.5) | 100 (2.2) | 0 | 0.150 |
| Region of residence |  |  | 10.5 | 0.348 |
| Stockholm | 542 (13.9) | 997 (24.4) |  |  |
| Uppsala | 1065 (27.3) | 869 (21.2) |  |  |
| Linkoping | 184 (4.7) | 285 (7.0) |  |  |
| Lund-Malmo | 867 (22.2) | 786 (19.2) |  |  |
| Gothenburg | 886 (22.7) | 652 (15.9) |  |  |
| Umeå | 362 (9.3) | 504 (12.3) |  |  |
| Degree of urbanisation |  |  | 10.5 | 0.011 |
| Most urbanised | 1318 (33.7) | 1364 (33.3) |  |  |
| Mid urbanised | 1694 (43.4) | 1777 (43.4) |  |  |
| Least urbanised | 894 (22.9) | 952 (23.3) |  |  |
| Moved municipality in last year | 79 (2.0) | 83 (2.0) | 10.5 | <0.001 |
| *Family structure* |  |  |  |  |
| Family position |  |  | 0.4 | 0.043 |
| Single | 1541 (35.3) | 1686 (37.2) |  |  |
| Single parent | 119 (2.7) | 111 (2.5) |  |  |
| Married, cohabiting, in a partnership | 2711 (62.0) | 2733 (60.3) |  |  |
| Divorce, separation, or loss of partner in last 3 years | 81 (1.9) | 113 (2.5) | 0.4 | 0.044 |
| *Education* |  |  |  |  |
| Education area |  |  | 3 | 0.107 |
| General education | 1455 (34.0) | 1704 (38.9) |  |  |
| Social sciences | 707 (16.5) | 654 (14.9) |  |  |
| Science, technology, agriculture, health and social care | 1861 (43.5) | 1746 (39.9) |  |  |
| Services | 260 (6.1) | 277 (6.3) |  |  |
| Education length |  |  | 1.4 | 0.114 |
| Primary education ≤9yrs | 1295 (29.8) | 1565 (35.1) |  |  |
| Secondary education ≤3yrs | 2073 (47.6) | 1959 (43.9) |  |  |
| Tertiary education </>3yrs | 983 (22.6) | 939 (21.0) |  |  |
| *Employment and income* |  |  |  |  |
| Occupational status |  |  | 1.7 | 0.294 |
| Senior Officials, managers and professionals | 632 (14.6) | 437 (9.8) |  |  |
| Small business owners and farmers | 361 (8.3) | 299 (6.7) |  |  |
| Supervisors, technicians and skilled manual workers | 295 (6.8) | 193 (4.3) |  |  |
| Office employees and skilled workers in trade, services, and care | 320 (7.4) | 216 (4.9) |  |  |
| Other manual worker | 457 (10.5) | 345 (7.8) |  |  |
| Retired or unemployed | 2274 (52.4) | 2957 (66.5) |  |  |
| At least one day of unemployment last 3 years | 383 (8.8) | 427 (9.4) | 0.4 | 0.023 |
| Individual disposable income per hundred SEK | 2270.3 [1573.0, 3161.8] | 1826.0 [1397.4, 2751.5] | 0.4 | 0.059 |
| Individual disposable income per hundred SEK by quintile |  |  | 0.4 | 0.315 |
| 1 | 502 (11.5) | 827 (18.3) |  |  |
| 2 | 615 (14.1) | 865 (19.1) |  |  |
| 3 | 841 (19.2) | 1010 (22.3) |  |  |
| 4 | 1143 (26.1) | 865 (19.1) |  |  |
| 5 | 1270 (29.1) | 963 (21.3) |  |  |

Supplementary Table 12: Baseline characteristics of female individuals who were and were not enrolled in the VALIDATE-SWEDEHEART trial

| Variable | Enrolled | Not enrolled | Missing | SMD |
| --- | --- | --- | --- | --- |
| N | 1567 | 2000 |  |  |
| *Demographics and migration* |  |  |  |  |
| Age (mean (SD)) | 70.8 (10.8) | 73.7 (11.3) | 0 | 0.254 |
| gender = Female | 1567 (100.0) | 2000 (100.0) | 0 | <0.001 |
| Country of birth |  |  | 0 | 0.304 |
| Sweden | 1382 (88.2) | 1571 (78.6) |  |  |
| Other Nordic countries | 96 (6.1) | 164 (8.2) |  |  |
| Europe excluding Nordics | 65 (4.1) | 164 (8.2) |  |  |
| North and South America | 7 (0.4) | 6 (0.3) |  |  |
| Other | 17 (1.1) | 95 (4.8) |  |  |
| Immigration in the 3 years prior | 5 (0.3) | 18 (0.9) | 0 | 0.075 |
| Region of residence |  |  | 10.4 | 0.404 |
| Stockholm | 154 (11.1) | 374 (20.7) |  |  |
| Uppsala | 422 (30.5) | 382 (21.1) |  |  |
| Linkoping | 62 (4.5) | 158 (8.7) |  |  |
| Lund-Malmo | 283 (20.4) | 377 (20.8) |  |  |
| Gothenburg | 327 (23.6) | 287 (15.8) |  |  |
| Umeå | 137 (9.9) | 233 (12.9) |  |  |
| Degree of urbanisation |  |  | 10.4 | 0.087 |
| Most urbanised | 444 (32.1) | 653 (36.1) |  |  |
| Mid urbanised | 602 (43.5) | 755 (41.7) |  |  |
| Least urbanised | 339 (24.5) | 403 (22.3) |  |  |
| Moved municipality in last year | 28 (2.0) | 23 (1.3) | 10.4 | 0.059 |
| *Family structure* |  |  |  |  |
| Family position |  |  | 0.1 | 0.140 |
| Single | 803 (51.2) | 1126 (56.4) |  |  |
| Single parent | 56 (3.6) | 98 (4.9) |  |  |
| Married, cohabiting, in a partnership | 708 (45.2) | 771 (38.6) |  |  |
| Divorce, separation, or loss of partner in last 3 years | 41 (2.6) | 75 (3.8) | 0.1 | 0.065 |
| *Education* |  |  |  |  |
| Education area |  |  | 3.1 | 0.192 |
| General education | 586 (38.1) | 908 (47.4) |  |  |
| Social sciences | 388 (25.2) | 427 (22.3) |  |  |
| Science, technology, agriculture, health and social care | 453 (29.4) | 456 (23.8) |  |  |
| Services | 112 (7.3) | 126 (6.6) |  |  |
| Education length |  |  | 1.9 | 0.193 |
| Primary education ≤9yrs | 541 (34.7) | 845 (43.5) |  |  |
| Secondary education ≤3yrs | 689 (44.3) | 787 (40.5) |  |  |
| Tertiary education </>3yrs | 327 (21.0) | 309 (15.9) |  |  |
| *Employment and income* |  |  |  |  |
| Occupational status |  |  | 0.7 | 0.272 |
| Senior Officials, managers and professionals | 109 (7.0) | 70 (3.5) |  |  |
| Small business owners and farmers | 42 (2.7) | 36 (1.8) |  |  |
| Supervisors, technicians and skilled manual workers | 5 (0.3) | 4 (0.2) |  |  |
| Office employees and skilled workers in trade, services, and care | 221 (14.1) | 161 (8.1) |  |  |
| Other manual worker | 38 (2.4) | 49 (2.5) |  |  |
| Retired or unemployed | 1147 (73.4) | 1659 (83.8) |  |  |
| At least one day of unemployment last 3 years | 60 (3.8) | 72 (3.6) | 0.1 | 0.012 |
| Individual disposable income per hundred SEK | 1503.7 [1243.0, 2096.5] | 1435.3 [1164.8, 1798.8] | 0.1 | 0.111 |
| Individual disposable income per hundred SEK by quintile |  |  | 0.1 | 0.196 |
| 1 | 462 (29.5) | 702 (35.2) |  |  |
| 2 | 423 (27.0) | 590 (29.6) |  |  |
| 3 | 291 (18.6) | 351 (17.6) |  |  |
| 4 | 255 (16.3) | 229 (11.5) |  |  |
| 5 | 136 (8.7) | 123 (6.2) |  |  |

Supplementary Table 13: Sensitivity analysis for the random forest predictive model for enrolment using complete case analysis

| **Covariates included** | **TASTE area under the ROC curve (95% CI)** | **VALIDATE-SWEDEHEART area under the ROC curve (95% CI)** |
| --- | --- | --- |
| Clinical | 0.58 (0.56 – 0.59) | 0.62 (0.61 – 0.63) |
| Socioeconomic | 0.56 (0.55 – 0.58) | 0.60 (0.58 – 0.61) |
| Clinical and socioeconomic | 0.58 (0.56 – 0.60) | 0.64 (0.62 – 0.65) |
